# Nanotherapeutic Approaches of Interleukin‐3 to Clear the α‐Synuclein Pathology in Mouse Models of Parkinson's Disease

**DOI:** 10.1002/advs.202405364

**Published:** 2024-09-03

**Authors:** Wenlong Zhang, Jian Ren, Liuyan Ding, Shaohui Zheng, Runfang Ma, Mengran Zhang, Yan Liu, Ruijing Liang, Yunlong Zhang

**Affiliations:** ^1^ Department of Neurology The First Affiliated Hospital of Guangzhou Medical University Guangzhou 510120 China; ^2^ Guangdong Key Laboratory of Nanomedicine CAS‐HK Joint Lab for Biomaterials Institute of Biomedicine and Biotechnology Shenzhen Institute of Advanced Technology Chinese Academy of Sciences Shenzhen 518055 China; ^3^ Westlake Laboratory of Life Sciences and Biomedicine Hangzhou 310024 China; ^4^ Key Laboratory of Neurological Function and Health School of Basic Medical Sciences Guangzhou Medical University Guangzhou 511436 China

**Keywords:** α‐synuclein, DA neuron, IL‐3, microglia, Parkinson's disease

## Abstract

Astrocyte‐microglia crosstalk is vital for neuronal survival and clearing aggregate accumulation in neurodegenerative diseases. While interleukin‐3 (IL‐3) has been reported to exert both protective and detrimental effects in neurodegenerative diseases, however, its role in α‐synuclein pathology remains unclear. In this study, it is found that astrocytic IL‐3 and microglial IL‐3R are positively responsive to α‐synuclein pathology in the brains of transgenic A53T Parkinson's disease (PD) mice and in an adeno‐associated virus (AAV)‐human α‐synuclein (AAV‐*h*α‐Syn)‐injected PD mouse model. Exogenous IL‐3 infusion reduces behavioral abnormities and nigrostriatal α‐synuclein pathology. Mechanistically, IL‐3 induces microglial phagocytosis of pathological α‐synuclein while simultaneously stimulating dopaminergic (DA) neurons to clear pathological α‐synuclein via induction of autophagy through the IFN‐β/Irgm1 pathway. Due to its limited efficiency in crossing the blood–brain barrier, a precise IL‐3 delivery strategy is developed by cross‐linking IL‐3 and RVG29 with PEG‐Linker (RVG‐modified IL‐3 nanogels—RVG‐IL3 NGs). Intravenous administration of RVG‐IL3 NGs shows efficient uptake by microglia and DA neurons within the brain. RVG‐IL3 NGs ameliorate motor deficits and pathological α‐synuclein by improving microglial and neuronal function in the AAV‐*h*α‐Syn mouse model of PD. Collectively, IL‐3 may represent a feasible therapeutic strategy for PD.

## Graphical abstract

This study uncovers that IL‐3 promotes microglia to phagocytize pathological α‐synuclein, and it stimulates dopaminergic neuron to clear pathological α‐synuclein via autophagy. IL‐3 is cross‐linked with acid inflammatory‐sensitive PEG‐Linker (called RVG‐IL3 NGs). RVG‐IL3 NGs are precisely delivered into microglia and dopaminergic neuron in the brain, and it ameliorates motor deficits and pathological α‐synuclein in mouse models of Parkinson’s disease.

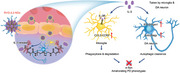

## 1 Introduction

Parkinson's disease (PD) is the second most common neurodegenerative disorder globally and is characterized by behavioral deficits, such as tremor and bradykinesia, as well as pathological hallmarks in the nigrostriatal system, such as loss of dopaminergic (DA) neurons and the presence of Lewy pathology, formed by α‐synuclein.^[^ ^1^ ^]^ Immune cells in the brain maintain or disrupt immune homeostasis, and neuroinflammation plays a fundamental role in the pathogenesis of PD.^[^ ^2^ ^]^ In the central nervous system (CNS), glia (e.g., microglia and astrocytes) are the predominant players in modulating brain immune function; accordingly, research interest in how these cells communicate has greatly increased.^[^ ^3^ ^]^ Activated microglia may be able to ignite astrocytes through the release of cytokines, such as interleukin‐1α (IL‐1α) and tumor necrosis factor (TNF), causing these reactive astrocytes to then contribute to neurodegenerative disorders.^[^ ^4^ ^]^ Blocking this conversion process ameliorates movement disorders, stops DA neuronal loss, and reduces α‐synuclein pathology in a PD mouse model.^[^ ^5^ ^]^ Astrocytes can engulf microglial debris, interrupting astrocytic APOE4, which alters microglial phagocytosis function.^[^ ^6^ ^]^ A previous study indicated that astrocytes that secrete complement C3 activate microglia, resulting in the release of inflammatory cytokines, and that this event exacerbates PD phenotypes.^[^ ^7^ ^]^ Recently, astrocytes have been reported to guide microglia to clear β‐amyloid (Aβ) and neurofibrillary tau in Alzheimer's disease (AD) by eliciting an astrocyte‐microglia interleukin‐3 (IL‐3) and its receptor (IL‐3R) cascade.^[^ ^8^ ^]^ Because astrocytes and microglia activation are also dependent on α‐synuclein burden,^[^ ^9^ ^]^ further examination of astrocyte‐microglia crosstalk as a benefit for PD is warranted.

IL‐3 is a multifunctional cytokine implicated in inflammatory and autoimmune diseases, such as sepsis and atherosclerosis.^[^ ^10^ ^]^ Although IL‐3 plays a specific role as a hematopoietic growth factor, it is also involved in neurodegenerative diseases. IL‐3 has been demonstrated to be protective in AD through the clearance of Aβ and tau.^[^ ^8^ ^]^ However, recent evidence has also revealed that astrocytic and T‐cell IL‐3 induce inflammatory responses and recruit IL‐3Rα^+^ myeloid cells to aggravate multiple sclerosis (MS).^[^ ^11^ ^]^ Thus, the role of IL‐3 signaling in PD remains unclear.

Most interleukins, like IL‐3, have a short plasma half‐life, poor transmembrane and cellular entry efficiency, and can also cause toxicity and immune risks when administered at high doses. These limitations have hindered their clinical application.^[^ ^12^ ^]^ Peripheral IL‐3 administration has been proven to poorly cross the blood–brain barrier (BBB).^[^ ^8^ ^]^ Thus, in the current study, we establish a nanogel system for IL‐3 delivery. Nanogels are three‐dimensional colloidal hydrogel nanoparticles consisting of physical or chemical crosslinked polymer networks. Their good biocompatibility, excellent ability to cross the BBB, advanced surface modifiability, stable loading capacity, and especially, efficient responsiveness to environmental stimuli, make nanogels promising drug delivery platforms for neurological disorders. Recently, several groups have developed nanogels system for delivering the drugs, such as l‐Dopa and activin B, for treating PD.^[^ ^13^ ^]^ In addition, modified nanogels have also been reported to inhibit Aβ aggregation in AD.^[^ ^14^ ^]^ These results indicate the therapeutic potentials of nanogels in the battle of against neurodegenerative diseases.

**Scheme 1:**
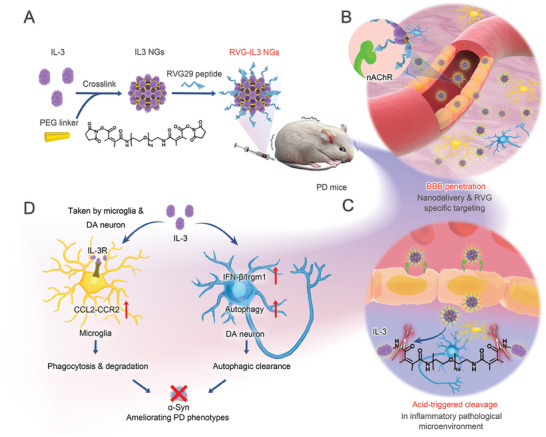
Schematic illustration of the preparation and application of RVG‐IL3 NGs for PD therapy. A) RVG‐IL3 NGs were fabricated by cross‐linking IL‐3 with an acid‐sensitive PEG‐Linker via bis‐N‐hydroxy succinimide (NHS) and further modified with RVG29. B) During blood circulation, RVG‐IL3 NGs showed enhanced brain accumulation via nano‐delivery and RVG‐specific identification of receptors (such as nAChR) on the BBB surface. C) The mechanism by which RVG‐IL3 NGs responded to the local acid inflammatory microenvironment and released IL‐3 in the PD brain. D) Mechanism of RVG‐IL3 NGs‐mediated α‐synuclein clearance.

In this study, astrocytic IL‐3 and microglial IL‐3R were found to be responsive to α‐synuclein pathology in the brains of transgenic A53T PD mice and in an adeno‐associated virus (AAV)‐human α‐synuclein (AAV‐*h*α‐Syn)‐injected PD mouse model. Exogenous IL‐3 infusion reduced behavioral abnormities and nigrostriatal α‐synuclein pathology. Mechanistically, IL‐3 induced microglial phagocytosis of pathological α‐synuclein while simultaneously stimulating DA neurons to clear pathological α‐synuclein via induction of autophagy through the interferon‐beta (IFN‐β)/ immune‐related GTPase M 1 (Irgm1) pathway. Notably, we developed an acid inflammatory‐sensitive PEG‐Linker that can cross‐link IL‐3 to create IL‐3 nanogels (IL3 NGs) and precisely deliver IL‐3 with RVG29, a 29‐residue peptide derived from the rabies virus glycoprotein (RVG), (hereafter referred to as RVG‐IL3 NGs) for PD therapy (**Scheme** **1**A). These RVG‐IL3 NGs with a uniform spherical morphology are able to release IL‐3 in response to acid inflammatory microenvironment stimulation while maintaining their natural biological activity. Additionally, the RVG‐IL3 NGs have the ability to enhance the blood circulation time of IL‐3, potentially due to their ability to evade the mononuclear phagocytosis system and avoid rapid clearance. These RVG‐IL3 NGs specifically target the nicotinic acetylcholine receptor (AchR) on brain vascular endothelial cells and nerve cells, resulting in increased cellular uptake by microglia and DA neurons in the brain (Scheme 1B,C). Furthermore, in a PD mouse model, RVG‐IL3 NGs were found to alleviate motor deficits and pathological α‐synuclein through microglia phagocytosis and neuronal autophagy (Scheme 1D). This study presents a promising strategy for the targeted delivery of IL‐3 to the CNS for PD treatment.

## 2 Results

### 2.1 IL‐3 Signaling Is Correlated with Nigrostriatal α‐Synuclein Pathology

**Figure 1:**
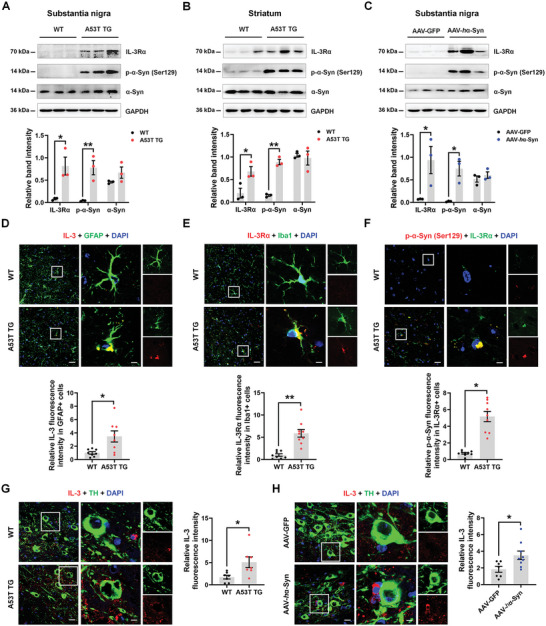
Astrocytic IL‐3 and microglial IL‐3R are positively correlated with α‐synuclein pathology. A,B) Representative blots and quantification showing the expression of IL‐3Rα, p‐α‐Syn (Ser129), and α‐Syn (α‐synuclein is abbreviated as α‐Syn in the legends) in the SN and striatum of WT and A53T transgenic mice (*n* = 3 per group). C) Representative blots and quantification showing the expression of IL‐3Rα, p‐α‐Syn (Ser129), and α‐Syn in the SN of AAV‐GFP and AAV‐*h*α‐Syn mice (*n* = 3 per group). D–F) Immunostaining and quantification of the colocalization between IL‐3 and GFAP, IL‐3Rα and Iba1, and p‐α‐Syn (Ser129) and IL‐3Rα in the SNpc of WT and A53T transgenic mice. *n* = 8–9 from 3 mice in each group. Scale bar, 20 µm. Magnified images are shown on the right. Scale bar, 6.5 µm. G) Immunostaining and quantification showing the intensity of IL‐3 in the SNpc of WT and A53T transgenic mice. *n* = 7 from 3 mice in each group. Scale bar, 25 µm. Magnified images are shown on the right. Scale bar, 10 µm. H) Immunostaining and quantification showing the intensity of IL‐3 in the SNpc of AAV‐GFP and AAV‐*h*α‐Syn mice. *n* = 7–9 from 3 mice in each group. Scale bar, 25 µm. Magnified images are shown on the right. Scale bar, 10 µm. Results are expressed as mean ± SEM. ^**^ *p* < 0.01, ^*^ *p* < 0.05 versus WT. A Student's *t*‐test was performed to determine statistical significance.

In this study, we focused on the IL‐3 signaling in the substantia nigra (SN) and striatum, which are regions that are susceptible to dysfunction in PD. Here, we identified elevated IL‐3Rα and phosphorylated α‐synuclein at serine 129, which is identified as a pathological hallmark in Lewy body disease,^[^ ^15^ ^]^ in the SN and striatum of transgenic A53T PD mice and the AAV‐*h*α‐Syn‐injected PD mouse model (**Figure** **1**A–C). Consistent with a previous study,^[^ ^8^ ^]^ IL‐3 was colocalized with glial fibrillary acidic protein (GFAP), a marker of astrocytes, and IL‐3Rα was colocalized with ionized calcium binding adaptor molecule 1 (Iba1), a marker of microglia in the substantia nigra pars compacta (SNpc) of A53T PD mice (Figure 1D,E), suggesting that astrocytes are the source of IL‐3 and that microglia express IL‐3Rα. We also detected the colocalization between phosphorylated α‐synuclein and IL‐3Rα in the SNpc of A53T PD mice (Figure 1F), suggesting IL‐3Rα‐positive microglia may engulf α‐synuclein. Since α‐synuclein aggregates are primarily present in DA neurons, we proved the pathological correlation between IL‐3 and DA neurons. We found high levels of IL‐3 surrounded tyrosine hydroxylase (TH)‐positive DA neurons in the SNpc of A53T PD mice and the AAV‐*h*α‐Syn‐injected PD mouse model (Figure 1G,H), which further verified that IL‐3 signaling was closely correlated with nigrostriatal α‐synuclein pathology.

### 2.2 Exogenous IL‐3 Infusion Attenuates PD Phenotypes via Modulation of Microglial Function

**Figure 2:**
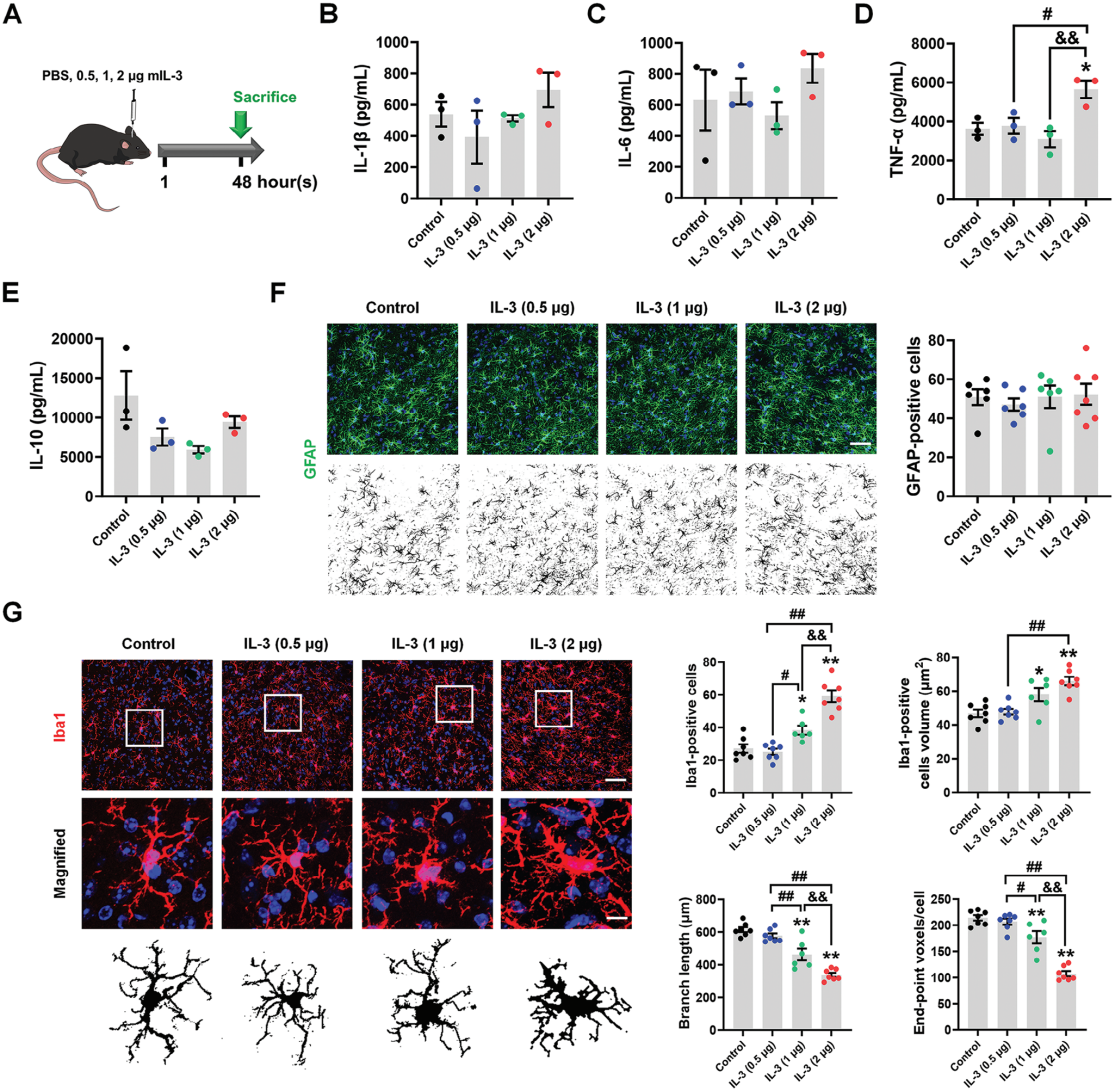
Effect of IL‐3 dosages on the midbrain immune response. A) Experimental design for 0.5, 1, 2 µg of IL‐3 administration in WT mice. B–E) The nigral levels of IL‐1β, IL‐6, TNF‐α, and IL‐10 were examined using ELISA kits. *n* = 3 per group. F) Immunostaining and quantification showing the number of GFAP‐positive cells in the SNpc. *n* = 6–7 from 3 mice in each group. Scale bar, 50 µm. G) Immunostaining and quantification showing the number and morphology of Iba1‐positive cells in the SNpc. *n* = 6–7 from 3 mice in each group. Scale bar, 50 µm. Magnified images are shown as below. Scale bar, 10 µm. Results are expressed as mean ± SEM. ^**^ *p* < 0.01, ^*^ *p* < 0.05 versus Control; ^##^ *p* < 0.01, ^#^ *p* < 0.05 versus 0.5 µg IL‐3; ^&&^ *p* < 0.01 versus 1 µg IL‐3. A one‐way ANOVA and a Tukey's test for post hoc comparisons were performed to determine statistical significance.

**Figure 3:**
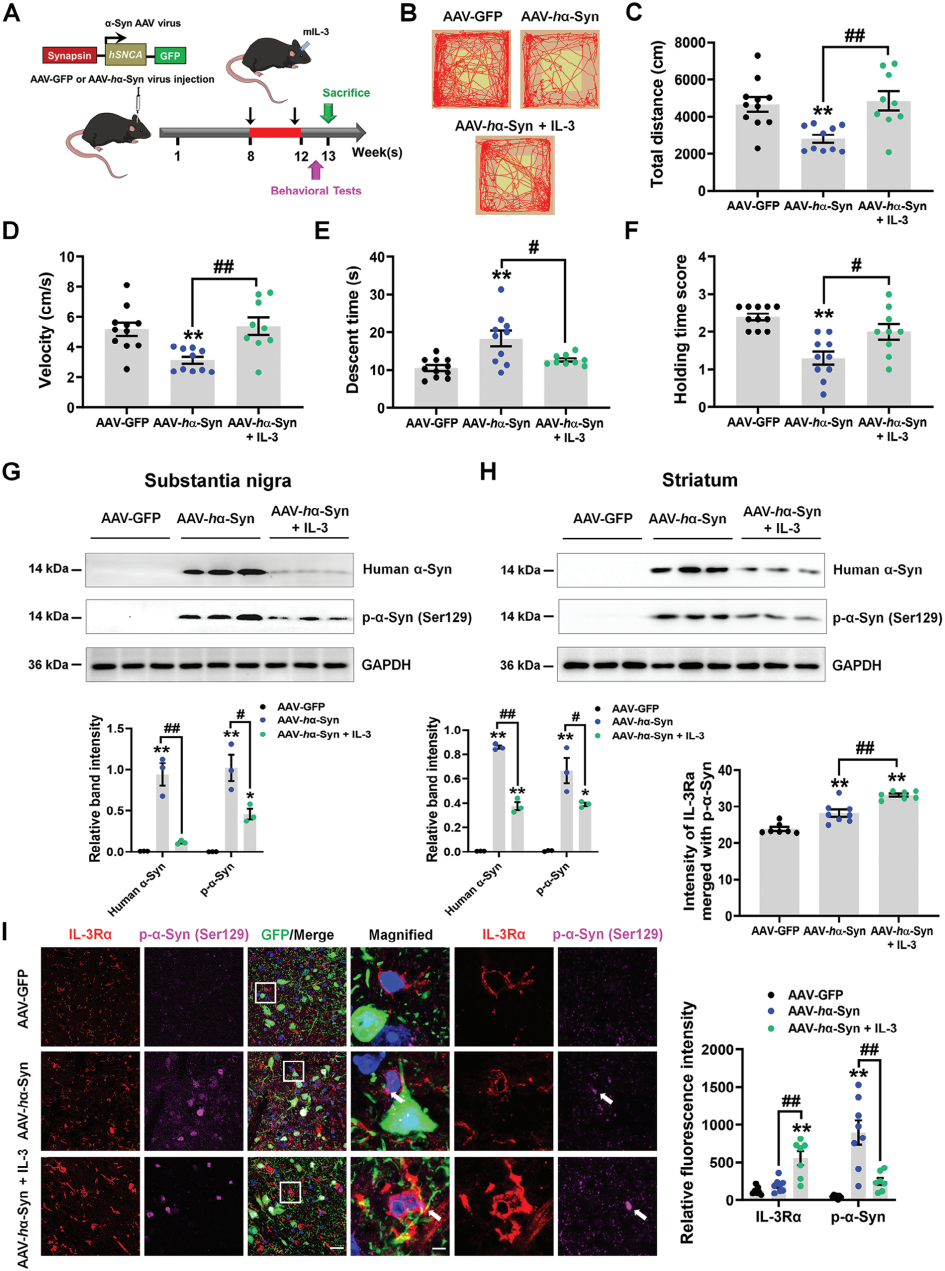
IL‐3 infusion ameliorates α‐synuclein pathology in AAV‐*h*α‐Syn mice. A) Experimental design for IL‐3 administration in AAV‐*h*α‐Syn mice. B–D) Representative traces, total distance traveled, and movement speed in the open field test. E) Descent time was used to examine bradykinesia in mice. F) Grasping test was used to examine the grip strength of mice. For AAV‐GFP, AAV‐*h*α‐Syn, and AAV‐*h*α‐Syn + IL‐3, *n  =  *11, 10, and 9, respectively. G,H) Representative blots and quantification showing the expression of human α‐Syn and p‐α‐Syn (Ser129) in the SN and striatum of AAV‐GFP and AAV‐*h*α‐Syn mice (*n* = 3 per group). I) Immunostaining and quantification of the intensity of IL‐3Rα merged with p‐α‐Syn, and relative fluorescent intensity of IL‐3Rα and p‐α‐Syn in the SNpc of AAV‐GFP, AAV‐*h*α‐Syn, and AAV‐*h*α‐Syn + IL‐3 mice (*n*  =  7, 8, and 7 from 3 mice in each group, respectively). Scale bar, 20 µm. Magnified images are shown on the right. Scale bar, 6.5 µm. Results are expressed as mean ± SEM. ^**^ *p* < 0.01, ^*^ *p* < 0.05 versus AAV‐GFP; ^##^ *p* < 0.01, ^#^ *p* < 0.05 versus AAV‐*h*α‐Syn. A one‐way ANOVA and a Tukey's test for post hoc comparisons were performed to determine statistical significance.

Because IL‐3 has been revealed to bind to microglial IL‐3Rα for clearance of Aβ and tau,^[^ ^8^ ^]^ and IL‐3Rα has been correlated with α‐synuclein pathology, we sought to investigate IL‐3/IL‐3R's effects on pathological α‐synuclein. Since IL‐3 itself is a pro‐inflammatory factor, we tested different concentrations of IL‐3 (0.5, 1, and 2 µg per mouse) on the brain immune response of wild‐type (WT) mice (**Figure** **2**A). After 48 h, a single dose of 2 µg IL‐3 significantly induced the production of TNF‐α but did not affect the levels of IL‐1β, IL‐6, and IL‐10 in the SN (Figure 2B–E). Though these dosages of IL‐3 did not affect the activation of astrocytes as indicated by the number of GFAP‐positive cells (Figure 2F), 1 and 2 µg of IL‐3 increased the Iba1‐positive cell numbers and volume, and decreased their process complexity and endpoint voxels in the SNpc (Figure 2G), suggesting microglia were activated under this condition. Considering 2 µg of IL‐3 may induce a pro‐inflammatory response and more severe microglial activation (Figure 2D,G), we thus chose 1 µg of IL‐3 to treat the mice, which was consistent with the administration dosage in a recently reported AD mouse model.^[^ ^8^ ^]^ Following the administration of mouse free IL‐3, the total distance traveled and movement speed of AAV‐*h*α‐Syn mice in the open field as well as their performance in pole‐climbing and grasping tests were significantly improved (**Figure** **3**A–F). In addition, IL‐3 infusion weakened exogenous human α‐synuclein and endogenous phosphorylated α‐synuclein in the SN and striatum of AAV‐*h*α‐Syn mice, compared with AAV‐*h*α‐Syn mice, suggesting that IL‐3 clears α‐synuclein pathology (Figure 3G,H). Interestingly, IL‐3 also promoted IL‐3Rα expression, which was colocalized with phosphorylated α‐synuclein in the SNpc of AAV‐*h*α‐Syn mice, compared with AAV‐*h*α‐Syn mice (Figure 3I).

**Figure 4:**
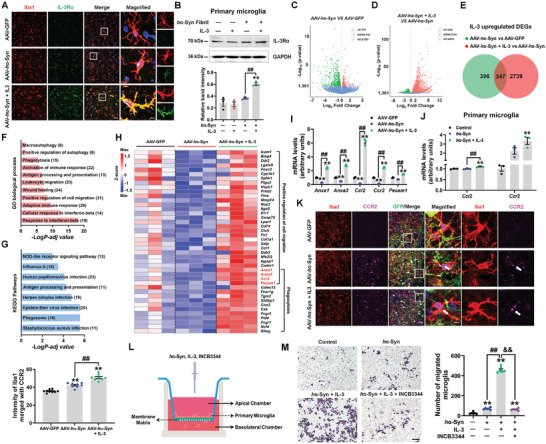
IL‐3 infusion promotes the migration and phagocytosis function of microglia in AAV‐*h*α‐Syn mice. A) Immunostaining showing Iba1 merged with IL‐3Rα in the SNpc of AAV‐GFP, AAV‐*h*α‐Syn, and AAV‐*h*α‐Syn + IL‐3 mice. Scale bar, 20 µm. Magnified images are shown on the right. Scale bar, 6.5 µm. B) Representative blots and quantification showing the expression of IL‐3Rα in IL‐3 and/or *h*α‐Syn fibril‐treated primary microglia (*n* = 3 per group). C,D) Volcano plot showing the DEGs between AAV‐*h*α‐Syn and AAV‐GFP, and AAV‐*h*α‐Syn + IL‐3 and AAV‐*h*α‐Syn. E) Venn diagram showing the number of IL‐3 upregulated DEGs in AAV‐*h*α‐Syn, which were decreased in AAV‐*h*α‐Syn compared with AAV‐GFP mice. F,G) GO and KEGG pathways enriched by these 347 DEGs. H) Hierarchical clustering of IL‐3 upregulated DEGs, which were enriched in “Positive regulation of cell migration” and “Phagocytosis”. Note that overlapped genes are highlighted in red. I) The mRNA expression levels of *Anxa1*, *Anxa3*, *Ccl2*, *Ccr2*, and *Pecam1* in the SN of AAV‐GFP, AAV‐*h*α‐Syn, and AAV‐*h*α‐Syn + IL‐3 mice (*n* = 3 per group). J) The mRNA expression levels of *Ccl2* and *Ccr2* in IL‐3 and/or *h*α‐Syn fibril‐treated primary microglia (*n* = 3 per group). K) Immunostaining and quantification of the intensity of Iba1 merged with CCR2 in the SNpc of AAV‐GFP, AAV‐*h*α‐Syn, and AAV‐*h*α‐Syn + IL‐3 mice (*n*  =  9, 8, and 8 from 3 mice in each group, respectively). Scale bar, 20 µm. Magnified images are shown on the right. Scale bar, 6.5 µm. L) Schematic illustration of Transwell assay for detecting microglia migration. M) The number of migrated microglia to the basolateral chamber upon IL‐3 treatment in *h*α‐Syn fibril‐treated primary microglia with or without CCR2 antagonist (INCB3344; *n* = 6 per group). Scale bar, 100 µm. Results are expressed as mean ± SEM. ^**^ *p* < 0.01 versus AAV‐GFP or control; ^##^ *p* < 0.01 versus AAV‐*h*α‐Syn or *h*α‐Syn fibril; ^&&^ *p* < 0.01 versus *h*α‐Syn fibril + IL‐3. A one‐way ANOVA and a Tukey's test for post hoc comparisons were performed to determine statistical significance.

IL‐3 enhanced microglial IL‐3Rα expression in the SNpc of AAV‐*h*α‐Syn mice and *h*α‐Syn fibril‐treated primary microglia (**Figures** **4**A,B and S1, Supporting Information). These data indicate that IL‐3 may activate the phagocytosis function of microglia. We then employed RNA‐sequencing (RNA‐seq) to identify underlying mechanisms (Figure S2, Supporting Information). The number of upregulated and downregulated genes after IL‐3 treatment in AAV‐*h*α‐Syn mice was much greater than the changes observed between the AAV‐*h*α‐Syn and AAV‐GFP groups (Figure 4C,D). Remarkably, IL‐3 increased 347 differentially expressed genes (DEGs), which were downregulated in AAV‐*h*α‐Syn versus AAV‐GFP (Figure 4E). Pathways enriched by these DEGs were primarily involved in the immune response, cell migration, phagocytosis, and macroautophagy (Figure 4F,G and Figures S3 and S4, Supporting Information). As our results indicated that IL‐3 may activate microglia to clear α‐synuclein, we then focused on DEGs related to microglial migration and phagocytosis. We found four genes (*Anxa1*, *Anxa3*, *Ccr2*, and *Pecam1*) that overlapped in these two pathways (highlighted in red in Figure 4H). Because the CC chemokine receptor 2 (CCR2) is the receptor for the chemokine C‐C motif ligand 2 (CCL2),^[^ ^16^ ^]^ we examined the expression of these five genes in the SN of AAV‐*h*α‐Syn mice treated with IL‐3. Notably, IL‐3 significantly increased *Ccl2* levels in AAV‐*h*α‐Syn mice and *h*α‐Syn fibril‐treated primary microglia (Figure 4I,J), which was consistent with IL‐3′s effects in an AD mouse model.^[^ ^8^ ^]^ Moreover, we found that IL‐3 increased the signal of CCR2 in microglia in the SNpc of AAV‐*h*α‐Syn mice (Figure 4K), suggesting that CCR2 is critical for IL‐3 activation of microglial IL‐3Rα. To test this possibility, we used a Transwell assay to test IL‐3′s effects on microglial migration. We seeded primary microglia on the membrane of the apical chamber with or without *h*α‐Syn fibril, IL‐3, and CCR2 antagonists (INCB3344; Figure 4L). Here, we observed that IL‐3 significantly induced microglia migration compared with the *h*α‐Syn fibril group, which was blocked by INCB3344 (Figure 4M). Though we did not observe that IL‐3 treatment altered the numbers of microglia and astrocytes in the SNpc compared with the AAV‐*h*α‐Syn mice (Figure S5, Supporting Information), our data indicated that IL‐3 modulates microglia migration and phagocytosis in the PD mouse model.

### 2.3 IL‐3 Infusion Promotes DA Neurons to Clear α‐Synuclein via Autophagy

**Figure 5:**
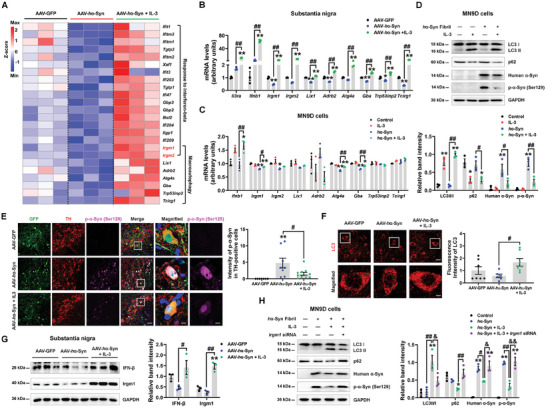
IL‐3 infusion induces neuronal autophagy in AAV‐*h*α‐Syn mice. A) Hierarchical clustering of IL‐3 upregulated DEGs, which were enriched in “Response to interferon‐beta” and “Macroautophagy”. Note that overlapped genes (*Irgm1* and *Irgm2*) are highlighted in red. B) The mRNA expression levels of *Il3ra*, *Ifnb1*, *Irgm1*, *Irgm2*, *Lix1*, *Adrb2*, *Atg4a*, *Gba*, *Trp53inp2*, and *Tcirg1* in the SN of AAV‐GFP, AAV‐*h*α‐Syn, and AAV‐*h*α‐Syn + IL‐3 mice (*n* = 3 per group). C) The mRNA expression levels of *Ifnb1*, *Irgm1*, *Irgm2*, *Lix1*, *Adrb2*, *Atg4a*, *Gba*, *Trp53inp2*, and *Tcirg1* in IL‐3 and/or *h*α‐Syn fibril‐treated MN9D cells (*n* = 3 per group). D) Representative blots and quantification showing the expression of LC3 II/I, p62, human α‐Syn, and p‐α‐Syn (Ser129) in IL‐3 and/or *h*α‐Syn fibril‐treated MN9D cells (*n* = 3 per group). E) Immunostaining and quantification of p‐α‐Syn intensity in TH‐positive cells in the SNpc of AAV‐GFP, AAV‐*h*α‐Syn, and AAV‐*h*α‐Syn + IL‐3 mice (*n*  =  7, 8, and 11 from 3 mice in each group, respectively). Scale bar, 20 µm. Magnified images are shown on the right. Scale bar, 6.5 µm. F) Immunostaining and quantification of LC3 intensity in the SNpc of AAV‐GFP, AAV‐*h*α‐Syn, and AAV‐*h*α‐Syn + IL‐3 mice (*n*  =  7, 6, and 7 from 3 mice in each group, respectively). Scale bar, 20 µm. Magnified images are shown on the bottom. Scale bar, 4 µm. G) Representative blots and quantification showing the expression of IFN‐β and Irgm1 in the SN of AAV‐GFP, AAV‐*h*α‐Syn, and AAV‐*h*α‐Syn + IL‐3 mice (*n* = 3 per group). H) Representative blots and quantification showing the expression of LC3 II/I, p62, human α‐Syn, and p‐α‐Syn (Ser129) upon IL‐3 treatment in *h*α‐Syn fibril‐treated MN9D cells in the presence of *Irgm1* siRNA (*n* = 3 per group). Results are expressed as mean ± SEM. ^**^ *p* < 0.01, ^*^ *p* < 0.05 versus AAV‐GFP or control; ^##^ *p* < 0.01, ^#^ *p* < 0.05 versus AAV‐*h*α‐Syn or *h*α‐Syn fibril; ^&&^ *p* < 0.01, ^&^ *p* < 0.05 versus *h*α‐Syn fibril + IL‐3. A one‐way ANOVA and a Tukey's test for post hoc comparisons were performed to determine statistical significance.

RNA‐seq data also indicated that IL‐3 promoted IFN‐β release and activated autophagy‐related pathways (Figure 4F,G). Neuronal IFN‐β typically induces autophagy and α‐synuclein clearance, and loss of neuronal IFN‐β‐IFNAR signaling causes Lewy body aggregation via autophagy impairment.^[^ ^17^ ^]^ Inspired by this evidence, we listed the DEGs enriched in IFN‐β and autophagy pathways and identified that *Irgm1* and *Irgm2* overlapped with IL‐3‐upregulated DEGs (highlighted in red in **Figure** **5**A). Interestingly, Irgm1 regulates autophagy and is the downstream target of IFN‐β signaling.^[^ ^18^ ^]^ Here, we verified our RNA‐seq data and found that the expression levels of *Il3ra* (encoding IL‐3Rα), *Ifnb1* (encoding IFN‐β), *Irgm1* (encoding Irgm1), *Irgm2* (encoding Irgm2), and other autophagy‐related genes were enhanced in the SN of AAV‐*h*α‐Syn mice upon administration of IL‐3 (Figure 5B). Furthermore, results from the stereotactic injection of different concentrations of IL‐3 suggested that 1 and 2 µg of IL‐3 significantly increased the microtubule‐associated protein light chain 3 (LC3) II/I ratio and decreased the p62/SQSTM1 expression in the SN, indicating that autophagy was activated (Figure S6, Supporting Information). In vitro results revealed that IL‐3 increased the expression of *Ifnb1* and *Irgm1* in *h*α‐Syn fibril‐treated DA MN9D cells but not in microglia (Figure 5C and Figure S7A, Supporting Information), suggesting that IL‐3 may enhance IFN‐β/Irgm1 signaling in neurons. IL‐3 increased LC3 II/I ratio and decreased p62/SQSTM1 expression; furthermore, it suppressed human α‐synuclein and phosphorylated α‐synuclein in *h*α‐Syn fibril‐treated MN9D cells (Figure 5D), suggesting that IL‐3 clears α‐synuclein via inducing neuronal autophagy. We further detected that IL‐3 decreased DA neuronal phosphorylated α‐synuclein while simultaneously increasing the expression levels of LC3 and IFN‐β/Irgm1 in SNpc of AAV‐*h*α‐Syn mice (Figure 5E–G). However, we did not find that IL‐3 alters the expression of IFN‐β/Irgm1 in *h*α‐Syn fibril‐treated primary microglia (Figure S7B, Supporting Information). To investigate whether Irgm1 was involved in the induction of autophagy by IL‐3, we designed a small interfering RNA (siRNA) targeting *Irgm1* (Figure S8A,B, Supporting Information; the second siRNA sequence was selected). Here, *Irgm1* knockdown abolished IL‐3‐induced increased LC3 II/I ratio and decreased the expression level of p62 in the *h*α‐Syn fibril‐treated MN9D cells (Figure 5H). In addition, *Irgm1* knockdown inhibited IL‐3′s effects on the clearance of human α‐synuclein and phosphorylated α‐synuclein in the *h*α‐Syn fibril‐treated MN9D cells (Figure 5H). Together, these pieces of evidence suggest that IL‐3 promotes the elimination of pathological α‐synuclein in DA neurons via IFN‐β/Irgm1‐induced autophagy.

### 2.4 Design of an IL‐3 Multiple Delivery System

**Figure 6:**
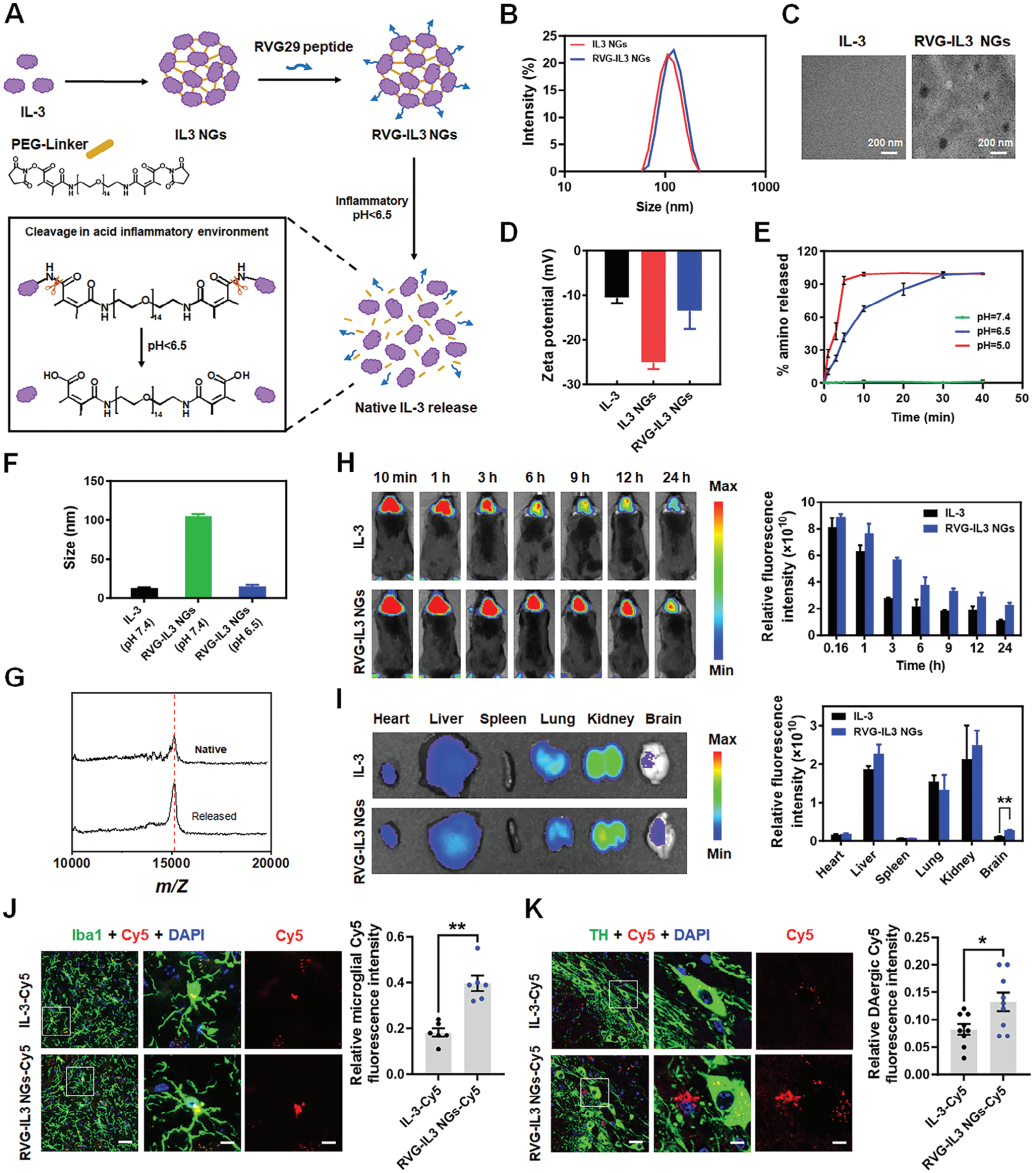
Preparation and characterization of RVG‐IL3 NGs. A) Schematic representation of the synthesis and cleavage of RVG‐IL3 NGs for native IL‐3 release in response to local acid inflammatory microenvironment. B) Size distribution analysis of IL3 NGs and RVG‐IL3 NGs. C) The transmission electron microscopy image of IL‐3 and RVG‐IL3 NGs. D) Zeta potential measurements of IL‐3, IL3 NGs, and RVG‐IL3 NGs. E) Percentage of exposed amino groups in DMA‐PEG‐DMA dispersed in solutions with varying pH and time. % amino exposed was calculated as: % amino exposed = Abs_340_ / (Abs_340,max –_ Abs_340,min_) × 100% (*n* = 3 per group). F) Size variation analysis of RVG‐IL3 NGs dispersed in PBS with pH 7.4 or 6.5. G) The mass spectrometry analysis of native and released IL‐3 tested via BRUKER Ultraflextreme. H) In vivo fluorescence tracking following intravenous injection of Cy5‐labeled IL‐3 and Cy5‐labeled RVG‐IL3 NGs in mice. Relative fluorescence intensity analysis of IVIS imaging of mice brains was shown in the right panel (*n* = 3 per group). I) Ex vivo fluorescence images of organs and brains and relative fluorescence intensity analysis of different tissues at 24 h post‐treatment with Cy5‐labeled IL‐3 or Cy5‐labeled RVG‐IL3 NGs (*n* = 3 per group). J) Distribution and quantification of Cy5‐labeled IL‐3 or Cy5‐labeled RVG‐IL3 NGs in Iba1‐positive cells in the SNpc. *n*  =  6 from 3 mice in each group, respectively. Scale bar, 20 µm. Magnified images are shown on the right. Scale bar, 6.5 µm. K) Distribution and quantification of Cy5‐labeled IL‐3 or Cy5‐labeled RVG‐IL3 NGs in TH‐positive cells in the SNpc. *n*  =  9 from 3 mice in each group, respectively. Scale bar, 20 µm. Magnified images are shown on the right. Scale bar, 6.5 µm. Results are expressed as mean ± SEM. ^**^ *p* < 0.01; ^*^ *p* < 0.05 versus free IL‐3. A Student's *t*‐test was performed to determine statistical significance.

Because peripheral IL‐3 administration does not efficiently cross the BBB,^[^ ^8^ ^]^ we aimed to design a novel IL‐3 delivery system to effectively target microglia and DA neurons. First, we synthesized an acid‐sensitive PEG‐Linker with bis‐N‐hydroxy succinimide (NHS; Figure S9A,B, Supporting Information). Due to the neuronal orientation property of RVG29,^[^ ^19^ ^]^ RVG‐IL3 NGs were prepared by cross‐linking IL‐3 and RVG29 with PEG‐Linker (**Figure** **6**A). The average hydrodynamic sizes of IL3 NGs and RVG‐IL3 NGs were 106 ± 3 and 122 ± 4 nm, respectively, with similar size distributions (Figure 6B). Transmission electron microscopy (TEM) images confirmed that the RVG‐IL3 NGs exhibited a uniform spherical morphology (Figure 6C). Figure 6D shows that the zeta potentials of IL‐3, IL3 NGs, and RVG‐IL3 NGs were −10 ± 1, −20 ± 2, and −13 ± 4 mV, respectively. To investigate the pH‐responsive fracture of the PEG‐Linker for the release of IL‐3 under different acidic conditions, we utilized the *o*‐phthalaldehyde (OPA) reagent in combination with a thiol reagent to quantitatively detect the exposed primary amines of DMA‐PEG‐DMA under various pH solutions (Figure S10A, Supporting Information). The PEG‐Linker exhibited high stability in a pH 7.4 solution but showed a rapid and sensitive response to fractures and exposure to the amino groups when dispersed in acidic solutions (pH 6.5, simulating the inflammatory pH, and pH 5.0, simulating the lysosomal pH) (Figure S10B–D, Supporting Information). The results also demonstrated that 100% of the amino groups were exposed within 30 min at pH 6.5 and within 10 min at pH 5.0 (Figure 6E). Therefore, we could infer that the RVG‐IL3 NGs can maintain stability in a neutral physiological environment and release IL‐3 upon entering the inflammatory environment in PD mice or the lysosomes of nerve cells. Furthermore, in order to assess the acid‐responsive cleavage performance of RVG‐IL3 NGs, PBS with pH 6.5 was used to simulate the local acid inflammatory microenvironment, and size changes of RVG‐IL3 NGs dispersed in PBS, with pH 6.5 or 7.4, were monitored via dynamic light scattering (DLS). The results in Figure 6F show that upon exposure to PBS with pH 6.5, the size of RVG‐IL3 NGs decreased significantly compared with that of IL‐3, demonstrating the acid‐responsive cleavage behavior of RVG‐IL3 NGs to release native IL‐3 in the local acid inflammatory microenvironment. IL‐3 released from the RVG‐IL3 NGs exhibited the expected molecular weight (Figure 6G), suggesting the release of intact IL‐3 without extensive residual chemical groups. We used the CCK8 assay to analyze the cell viability of microglia and the SH‐SY5Y cells treated with free IL‐3 and RVG‐IL3 NGs. Figure S11 (Supporting Information) showed that the RVG‐IL3 NGs showed good biocompatibility on microglia and SH‐SY5Y cells. To study the brain‐targeted delivery efficiency of protein nanogels, we evaluated the enrichment rate in the brains of PD mice with Cy5‐labeled IL‐3 and Cy5‐labeled RVG‐IL3 NGs at different time points (10 min, 1 h, 3 h, 6 h, 9 h, 12 h, and 24 h), post‐administration, via the In Vivo Imaging System (IVIS) imaging. The results showed that compared with the IL‐3 group, treatment with RVG‐IL3 NGs showed a more rapid and efficient accumulation in the brain at 1 h post‐administration (Figure 6H). Furthermore, the fluorescence intensity of the brain regions of mice treated with RVG‐IL3 NGs remained higher than that of those treated with free IL‐3 within 24 h (Figure 6I). Biodistribution results show the enhanced accumulation of RVG‐IL3 NGs in brains, due to long blood circulation of nanocarriers, and the unique property of RVG to specifically recognize receptors on the BBB surface (nAChR) to deliver RVG‐IL3 NGs into the CNS. Compared with Cy5‐labeled IL‐3, Cy5‐labeled RVG‐IL3 NGs were primarily distributed in the microglia and DA neurons, rather than astrocytes (Figure 6J,K and Figure S12, Supporting Information). In addition, we also conducted flow cytometry to compare the uptake efficiency of Cy5‐labeled IL‐3 and Cy5‐labeled RVG‐IL3 NGs in microglia, MN9D cells, and astrocytes. We found an increased uptake of Cy5‐labeled RVG‐IL3 NGs in microglia and MN9D cells compared with Cy5‐labeled IL‐3 (Figure S13A,B, Supporting Information). The signals showed no significance between the Cy5‐labeled IL‐3 and the Cy5‐labeled RVG‐IL3 NGs in astrocytes (Figure S13C, Supporting Information). These results further confirmed our in vivo biodistribution results. Hence, we verified the efficacy of the IL‐3 delivery system for targeting microglia and DA neurons.

### 2.5 RVG‐IL3 NGs Improve Movement Dysfunction and Pathological Abnormities in AAV‐*h*α‐Syn Mice

**Figure 7:**
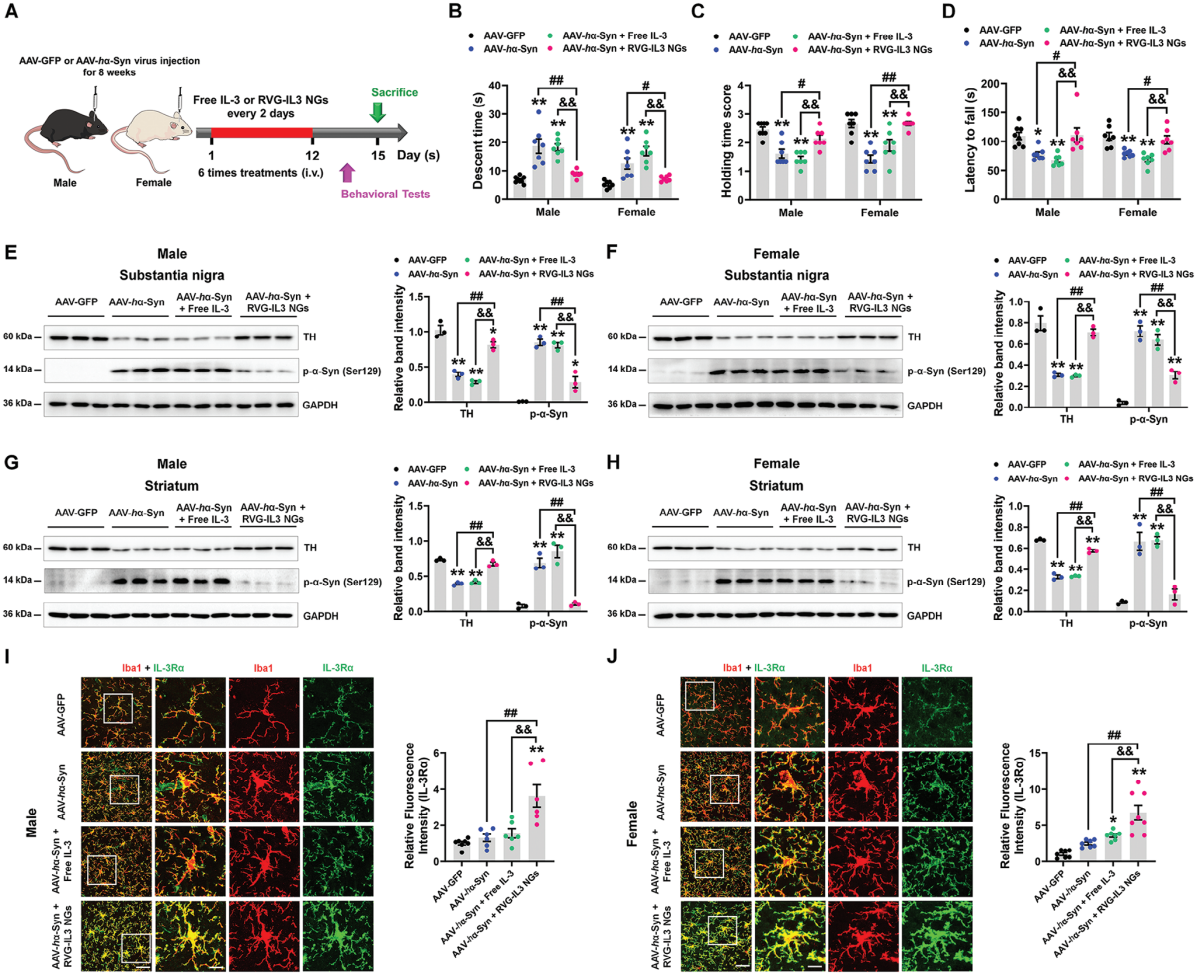
RVG‐IL3 NGs improve motor deficits and pathological abnormalities in AAV‐*h*α‐Syn mice. A) Experimental design for detecting the effects of free IL‐3 or RVG‐IL3 NGs in male and female AAV‐*h*α‐Syn mice. B) Descent time was used to examine bradykinesia in mice. C) Grasping test was used to examine the grip strength of mice. D) Rotarod test was used to examine the motor coordination of mice. For male AAV‐GFP, AAV‐*h*α‐Syn, AAV‐*h*α‐Syn + free IL‐3, and AAV‐*h*α‐Syn + RVG‐IL3 NGs mice, *n*  =  8, 7, 7, and 7, respectively; for female mice in the above groups, *n* = 7 per group. E–H) Representative blots and quantification showing the expression of TH and p‐α‐Syn (Ser129) in the SN and striatum of male and female AAV‐GFP, AAV‐*h*α‐Syn, AAV‐*h*α‐Syn + free IL‐3, and AAV‐*h*α‐Syn + RVG‐IL3 NGs mice (*n* = 3 per group). I,J) Immunostaining and quantification of IL‐3Rα intensity in the SNpc of AAV‐GFP, AAV‐*h*α‐Syn, AAV‐*h*α‐Syn + free IL‐3, and AAV‐*h*α‐Syn + RVG‐IL3 NGs mice. For male AAV‐GFP, AAV‐*h*α‐Syn, AAV‐*h*α‐Syn + free IL‐3, and AAV‐*h*α‐Syn + RVG‐IL3 NGs mice, *n*  =  7, 6, 6, and 6 from 3 mice in each group, respectively; for female AAV‐GFP, AAV‐*h*α‐Syn, AAV‐*h*α‐Syn + free IL‐3, and AAV‐*h*α‐Syn + RVG‐IL3 NGs mice, *n*  =  7, 7, 7, and 8 from 3 mice in each group, respectively. Scale bar, 40 µm. Magnified images are shown on the right. Scale bar, 13 µm. Results are expressed as mean ± SEM. ^**^ *p* < 0.01, ^*^ *p* < 0.05 versus AAV‐GFP; ^##^ *p* < 0.01, ^#^ *p* < 0.05 versus AAV‐*h*α‐Syn; ^&&^ *p* < 0.01 versus AAV‐*h*α‐Syn + free IL‐3. A one‐way ANOVA and a Tukey's test for *post hoc* comparisons were performed to determine statistical significance.

PD displays sex‐specific characteristics,^[^ ^20^ ^]^ so to comprehensively examine the effects of IL‐3 in PD across sexes, we assessed the effects of intravenous injection of free IL‐3 and RVG‐IL3 NGs in both male and female AAV‐*h*α‐Syn mice (**Figure** **7**A). Here, compared with free IL‐3, RVG‐IL3 NGs shortened the descent time and reinforced the grip strength and motor coordination of male and female AAV‐*h*α‐Syn mice (Figure 7B–D). In addition, compared with free IL‐3, RVG‐IL3 NGs increased the nigrostriatal expression of TH, the rate‐limiting enzyme in the synthesis of dopamine, in male and female AAV‐*h*α‐Syn mice (Figure 7E–H and Figures S14 and S15, Supporting Information). RVG‐IL3 NGs also significantly reduced phosphorylated α‐synuclein in the SN and striatum of male and female AAV‐*h*α‐Syn mice compared with free IL‐3 (Figure 7E–H). These data suggest that RVG‐IL3 NGs reversed behavioral deficits and DA neuronal loss, and reduced α‐synuclein pathology in the PD mouse model.

**Figure 8:**
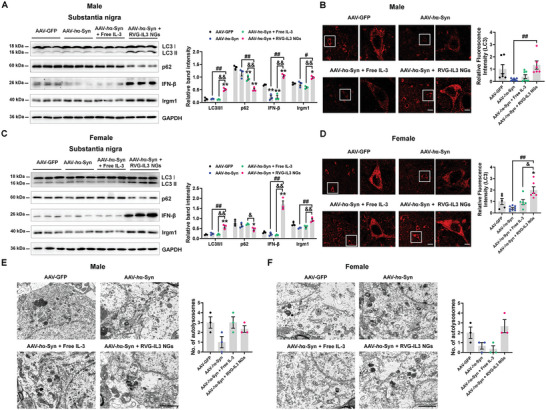
RVG‐IL3 NGs enhance neuronal autophagy in AAV‐*h*α‐Syn mice. A) Representative blots and quantification showing the expression of LC3 II/I, p62, IFN‐β, and Irgm1 in the SN of male AAV‐GFP, AAV‐*h*α‐Syn, AAV‐*h*α‐Syn + free IL‐3, and AAV‐*h*α‐Syn + RVG‐IL3 NGs mice (*n* = 3 per group). B) Immunostaining and quantification of LC3 intensity in the SNpc of male AAV‐GFP, AAV‐*h*α‐Syn, AAV‐*h*α‐Syn + free IL‐3, and AAV‐*h*α‐Syn + RVG‐IL3 NGs mice (*n*  =  6, 8, 7, and 6 from 3 mice in each group, respectively). Scale bar, 20 µm. Magnified images are shown on the right. Scale bar, 4 µm. C) Representative blots and quantification showing the expression of LC3 II/I, p62, IFN‐β, and Irgm1 in the SN of female AAV‐GFP, AAV‐*h*α‐Syn, AAV‐*h*α‐Syn + free IL‐3, and AAV‐*h*α‐Syn + RVG‐IL3 NGs mice (*n* = 3 per group). D) Immunostaining and quantification of LC3 intensity in the SNpc of female AAV‐GFP, AAV‐*h*α‐Syn, AAV‐*h*α‐Syn + free IL‐3, and AAV‐*h*α‐Syn + RVG‐IL3 NGs mice (*n*  =  5, 8, 6, and 6 from 3 mice in each group, respectively). Scale bar, 20 µm. Magnified images are shown on the right. Scale bar, 4 µm. E,F) Representative ultrastructural images and quantitative analysis of autolysosomes in the SN of male and female AAV‐GFP, AAV‐*h*α‐Syn, AAV‐*h*α‐Syn + free IL‐3, and AAV‐*h*α‐Syn + RVG‐IL3 NGs mice (*n*  =  3 per group). Scale bars, 2 µm. Results are expressed as mean ± SEM. ^**^ *p* < 0.01, ^*^ *p* < 0.05 versus AAV‐GFP; ^##^ *p* < 0.01, ^#^ *p* < 0.05 versus AAV‐*h*α‐Syn; ^&&^ *p* < 0.01, ^&^ *p* < 0.05 versus AAV‐*h*α‐Syn + free IL‐3. A one‐way ANOVA and a Tukey's test for *post hoc* comparisons were performed to determine statistical significance.

We also examined the time‐dependent activation of Iba1‐positive cells after RVG‐IL3 NGs treatment in AAV‐*h*α‐Syn mice. After three administrations, compared with the AAV‐*h*α‐Syn group, the RVG‐IL3 NGs further enlarged the number of Iba1‐positive cells (Figure S16, Supporting Information). RVG‐IL3 NGs treatment for six times decreased the microglial numbers; however, it further induced the phagocytic activation of microglia (Figure S16, Supporting Information). Consistent with IL‐3 infusion results, RVG‐IL3 NGs treatment for six times enhanced microglial IL‐3Rα expression (Figure 7I,J), revealing that RVG‐IL3 NGs induced microglial phagocytosis. In addition, we also examined the phagocytic pathological α‐synuclein in microglia in vitro. We found an accumulation of phosphorylated α‐synuclein in the *h*α‐Syn fibril and free IL‐3 treatment groups (Figure S17, Supporting Information). Furthermore, the RVG‐IL3 NGs highly phagocytosed phosphorylated α‐synuclein compared with the free IL‐3 (Figure S17, Supporting Information), suggesting that the RVG‐IL3 NGs enhanced the ability of microglia to phagocytic pathological α‐synuclein. Compared with free IL‐3, RVG‐IL3 NGs increased LC3 II/I ratio and LC3 expression and decreased p62 expression in the SN of male and female AAV‐*h*α‐Syn mice (**Figure** **8**A–D), suggesting that autophagy was activated. IFN‐β/Irgm1 signaling was also involved in RVG‐IL3 NGs‐induced autophagy activation in the SN of male and female AAV‐*h*α‐Syn mice (Figure 8A,C). In addition, we found increased numbers of autolysosomes in the SN of male and female AAV‐*h*α‐Syn mice upon treatment with RVG‐IL3 NGs (Figure 8E,F), although the difference was not significant. Collectively, these data indicate that RVG‐IL3 NGs improve the PD phenotype via modulation of microglial and neuronal function.

## 3 Discussion

As a pleiotropic cytokine, IL‐3 shows diverse effects in neurodegenerative diseases. While IL‐3 elicits inflammation and induces neuronal degeneration in AD, MS, and amyotrophic lateral sclerosis,^[^ ^11^, ^21^ ^]^ astrocytic IL‐3 may activate microglia to engulf aggregates of Aβ and tau.^[^ ^8^, ^22^ ^]^ Furthermore, IL‐3 protects DA neurons against 6‐hydroxydopamine‐mediated neurotoxicity by diminishing pro‐inflammatory response and increasing the expression of the anti‐apoptotic protein Bcl‐xL.^[^ ^23^, ^24^ ^]^ In this study, we showed that IL‐3 promotes the phagocytosis of microglia and autophagy of DA neurons to clear intracellular pathological α‐synuclein and shield against DA neuron death. Importantly, we established an effective and precise delivery system for IL‐3 intervention in PD.

In the CNS, microglia phagocytose aggregates, such as Aβ, tau, and α‐synuclein; neuronal and astrocytic debris; and myelin sheaths to maintain the microenvironment.^[^ ^8^, ^25^ ^]^ Astrocyte‐microglia crosstalk is critical for modulating microglial function, and IL‐3 seems to link this crosstalk for aggregate clearance. Here, we found that astrocytic IL‐3 and microglial IL‐3R were positively correlated with α‐synuclein pathology. Exogenous IL‐3 restored behavioral impairment and reduced pathological α‐synuclein. Furthermore, we found that IL‐3 may activate microglial IL‐3Rα via the CCL2/CCR2 signaling pathway. Normally, astrocytes and microglia are the source of CCL2; after release, it can bind with its microglial receptor (CCR2) to induce inflammation and migration.^[^ ^26^ ^]^ We found that IL‐3 increased microglial CCR2 expression and that blockade of CCR2 abolished IL‐3′s effects on α‐synuclein clearance. Furthermore, we hypothesize that IL‐3 may induce astrocytes or microglia to secrete CCL2, thus prompting microglia to migrate toward α‐synuclein and phagocytose it. However, further evidence is needed to indicate the role of astrocyte‐microglia crosstalk in this process.

Another finding of this study is that IL‐3 promotes neuronal autophagy for α‐synuclein clearance. In addition to astrocytes, neurons are a source of IL‐3, with IL‐3R also expressed.^[^ ^27^ ^]^ We identified that IL‐3 can induce neuronal autophagy and that this effect relies on the activation of IFN‐β/Irgm1 signaling. IFN‐β is critical for neuronal survival and neurite outgrowth; furthermore, neuronal IFN‐β deficiency has been revealed to damage autophagy and induce α‐synuclein aggregation,^[^ ^17^ ^]^ with the downstream mechanisms involved in the regulation of mitophagy and the miR1‐TBC1D15‐Rab7 pathway.^[^ ^17^, ^28^ ^]^ In the current study, we found that the target of IFN‐β, Irgm1, was a key regulator of autophagy, which was consistent with previous research.^[^ ^18^ ^]^ IL‐3 induced IFN‐β/Irgm1 signaling to engulf pathological α‐synuclein, thus preventing DA neuronal loss and motor deficits.

IL‐3 is a pro‐inflammatory factor, and the dosage of IL‐3 determines its pro‐inflammatory effect and therapeutic effect. Previously, IL‐3 was pumped to the lateral ventricle at a rate of 1 µg/day, and this dosage of IL‐3 mobilized microglia to Aβ plagues, although the intraperitoneally injection of IL‐3 for 10 weeks showed no therapeutic effects.^[^ ^8^ ^]^ Seven days of infusion of IL‐3 (64 or 320 ng/d) into the lateral ventricles prevents ischemia‐induced neuronal degeneration in a dose‐dependent manner.^[^ ^24^ ^]^ In addition, subcutaneous injection of IL‐3 at a dose of 10 µg kg^−1^ body weight for seven times attenuated stab wound‐induced brain injury in rats.^[^ ^29^ ^]^ By referring to the literature, we tested different concentrations of IL‐3 (0.5, 1, 2 µg per mouse). The 1 and 2 µg of IL‐3 doses promoted the autophagy; however, a single dose of 2 µg induced the production of TNF‐α and severe microglial activation. In addition, we also examined the time‐dependent activation of Iba1‐positive cells after RVG‐IL3 NGs treatment in the AAV‐*h*α‐Syn mice. Intermediate IL‐3 administration (three times) further induced the microglial number, while RVG‐IL3 NGs treatment for six times suppressed it. These results suggest that an excessive or short‐time accumulation of IL‐3 could cause a reactive pro‐inflammatory response. However, after full clearance of pathological α‐synuclein, the neuroinflammation was suppressed by IL‐3. This phenomenon reflects the dynamic brain reaction facing IL‐3 administration. Right now, we still do not know the paradigm of how IL‐3 changes the microglia from inflammatory to phagocytic states, and this requires further study.

To precisely deliver IL‐3 for PD therapy, we established an advanced delivery system (named RVG‐IL3 NGs) by cross‐linking IL‐3 with an acid‐sensitive PEG‐Linker via NHS and further modification by RVG29. As we previously reported,^[^ ^19^ ^]^ RVG29 is a 29‐residue peptide derived from the rabies virus glycoprotein that has been shown to specifically bind to neuronal cells expressing AchR, allowing RVG29‐modified IL3 NGs to cross the BBB through receptor‐mediated endocytosis. In the inflammatory pathological microenvironment, IL‐3 was released. Because RVG29 also guides particles to DA neurons and microglia,^[^ ^19^ ^]^ our system eventually targets these two cells. RVG‐IL3 NGs were observed to ameliorate behavioral deficits, clear α‐synuclein pathology, and protect DA neurons from death. Mechanistically, RVG‐IL3 NGs enhanced microglial IL‐3R signaling and activated neuronal autophagy.

Classically, movement disorders, such as tremor and bradykinesia, are the behavioral phenotypes of PD. Non‐motor symptoms, such as memory deficits and sleep disorders, are increasingly reported in PD patients.^[^ ^20^ ^]^ In this study, we primarily focused on the effects of IL‐3 on the motor function of PD mouse models; thus, we chose the pole‐climbing test, open field test, and rotarod test. Nevertheless, in the future, we need to clarify whether IL‐3 can rescue the memory ability of PD mouse models and further explore its potential use in treating neurodegenerative diseases.

In conclusion, the current study found that microglia migration and phagocytosis, as well as neuronal autophagy, contribute to the effects of IL‐3 on α‐synuclein clearance, neuronal survival, and behavioral improvement in a PD mouse model. Thus, IL‐3 may represent a promising therapeutic agent for the treatment of PD.

## 4 Experimental Section

### Reagents

Free mouse IL‐3 was purchased from PeproTech (Cranbury, NJ). Anti‐IL‐3 (ab167159), LC3 II/I (ab48394), α‐synuclein (ab138501), and p62 (ab56416) antibodies were purchased from Abcam (Cambridge, MA). Anti‐phospho‐α‐synuclein (Ser129; #23706), α‐synuclein (#4179), IFN‐β1 (#97450), Irgm1 (#71950), Iba1 (#17198), and LC3A/B (#12741) antibodies were purchased from Cell Signaling Technology (Danvers, MA). Anti‐GFAP (GA5, MAB360) antibody was purchased from Millipore (Burlington, MA). Anti‐IL‐3Rα (C‐5, sc‐74522) and TH (F‐11, sc‐25269) antibodies were purchased from Santa Cruz Biotechnology (Dallas, TX). Anti‐GAPDH (60004‐1) antibody was purchased from Proteintech (Rosemont, IL). Goat anti‐rabbit IgG (H + L) secondary antibody, DyLight 488 (35552), was purchased from Invitrogen (Waltham, MA). DyLight 488 goat anti‐mouse IgG (H + L; GAM4882), DyLight 594 goat anti‐mouse IgG (H + L; GAM5942), and DyLight 594 goat anti‐rabbit IgG (H + L; GAR5942) antibodies were purchased from Multi Sciences (Hangzhou, China). INCB3344 (HY‐50674) was purchased from MedChem Express (Princeton, NJ). In addition, 2, 3‐dimethylmaleic anhydride (DMA), amine‐polyethylene glycol‐amine (NH_2_‐PEG‐NH_2_, molecular weight *M*_w_ = 600), 4‐dimethylaminopyridine (DMAP), N, N′‐disuccinimidyl carbonate (DSC), and extra dry N, N′‐dimethylformamide (DMF) were obtained from Energy Chemical (Shanghai, China). N‐hexane, dichloromethane, methanol, dimethyl sulfoxide (DMSO), triethylamine (TEA), and isopropyl alcohol were provided by Chemical Reagent (Shanghai, China). The mouse IL‐1β enzyme‐linked immunosorbent assay (ELISA) Kit (#88‐7013), mouse IL‐6 ELISA Kit (#88‐7064), mouse TNF‐α ELISA Kit (#88‐7324), and the mouse IL‐10 ELISA Kit (#88‐7105) were purchased from Thermo Fisher Scientific (Waltham, MA, USA).

### Cell Culture and Drug Treatment

According to methods described in a recent study,^[^ ^30^ ^]^ primary microglia were obtained from C57BL/6J mice at postnatal days 1 and 2 and were cultured in Dulbecco's modified Eagle's medium/F12 (DMEM/F12, GIBCO, Carlsbad, CA) supplemented with 10% fetal calf serum (FCS; GIBCO, Carlsbad, CA) and GM‐CSF at 37 °C in 5% CO_2_. DA MN9D cells were purchased from the American Type Culture Collection (ATCC, Manassas, VA) and were cultured in Roswell Park Memorial Institute 1640 medium (GIBCO, Carlsbad, CA) supplemented with 10% FCS and 1% penicillin/streptomycin at 37 °C in 5% CO_2_. Primary astrocytes were obtained from the cerebral cortex of newborn C57BL/6J pups according to a recent work.^[^ ^7^, ^31^ ^]^ Astrocytes and SH‐SY5Y cells were cultured in DMEM/F12 supplemented with 10% FCS and 1% penicillin/streptomycin at 37 °C in 5% CO_2_. To determine the effects of IL‐3 on α‐synuclein clearance, microglia or MN9D cells were treated with *h*α‐Syn fibril (1 µg mL^−1^) and IL‐3 (20 ng mL^−1^) for 24 h.

### *Irgm1* siRNA Construction and Transfection

Three siRNA targeting *Irgm1* sequences (siRNA‐1, 5′‐ GAACCCTTAAAGACCCTTT‐3′; siRNA‐2, 5′‐ CCAAGAACATCCTAGAGAA‐3′; and siRNA‐3, 5′‐ GAAAGTGTACCGACTGATA‐3′) were designed and synthesized by RiboBio Co., Ltd. (Guangzhou, China), and negative‐control siRNA was also provided by RiboBio Co., Ltd. Lipo8000 Transfection Reagent (Beyotime Biotechnology, Shanghai, China) was used to transfect the siRNA, as reported recently.^[^ ^7^ ^]^

### Animals and Drug Treatments

Adult (10‐week‐old, 20–25 g of weight) male and female WT C57BL/6J mice were purchased from SPF Biotechnology Co., Ltd. (Beijing, China). The male homozygous hSNCA*A53T‐Tg mice (10‐month‐old, 35 g of weight, also called A53T TG mice) were purchased from the Shanghai Model Organisms Center, Inc. (Shanghai, China), and the age‐ and gender‐matched mice were used as a control. Mice were group‐housed under standard conditions with free access to food and water. The animal experimental protocols complied with the Institutional Animal Care and Use Committee of Guangzhou Medical University (Approval No. GY2020‐041) and National Institute of Health guidelines on the care and use of animals (NIH Publications No. 8023, revised 1978).

WT and A53T TG mice (*n* = 6 per group) were used to examine the expression profiles of IL‐3, IL‐3R, phosphorylated α‐synuclein, and α‐synuclein in the SNpc and striatum. In this study, a PD mouse model was constructed by stereotaxically injecting AAV‐GFP or AAV‐*h*α‐Syn‐GFP into the bilateral SNpc (Bregma AP, −3.0 mm, ML, ±1.3 mm, DV, −4.7 mm) of WT mice, as described in previous research.^[^ ^32^ ^]^ Mice were tested 8 weeks following surgery. To test the dosage of IL‐3, a single dose of 0.5, 1, and 2 µg were stereotaxically administrated to the WT mice. After 48 h, the samples were collected for immunoblotting and immunostaining. *N =* 6 per group, and PBS was used as the control. To examine the effects of IL‐3 on α‐synuclein clearance, mice were given mouse free IL‐3 1 µg d^−1^ every 2 d for 4 weeks through an intravenous cannula. *N =  *11, 10, and 9 for the AAV‐GFP, AAV‐*h*α‐Syn, and AAV‐*h*α‐Syn + IL‐3 groups, respectively. To test the effects of RVG‐IL3 NGs, male and female PD mice were intravenously administered with free IL‐3 and RVG‐IL3 NGs every 2 d, for a total of six doses. Then the behavioral tests and molecular biology experiments, such as immunoblotting and immunostaining, were performed. *N* =  11, 10, 10, and 10 for the male AAV‐GFP, AAV‐*h*α‐Syn, AAV‐*h*α‐Syn + free IL‐3, and AAV‐*h*α‐Syn + RVG‐IL3 NGs mice, respectively. *N* = 10 per group for the female mice in the above groups. The substantia nigra for the western blot in this study was isolated using brain matrices (RWD Life Science, Shenzhen, China) according to the its coordinate in the Mouse Brain Atlas.

### Synthesis of Acid Inflammatory‐Sensitive PEG‐Linker

To synthesize disulfide‐containing 2,3‐dimethylmaleic‐polyethylene glycol‐2,3‐dimethylmaleic (DMA‐PEG‐DMA) crosslinkers, 300 mg of NH_2_‐PEG‐NH_2_ was dissolved in dichloromethane, followed by the addition of DMA (300 mg) and three to five drops of TEA. The mixture was then stirred for 4–6 h and subsequently suspended to obtain a crude product. Finally, the product was purified using silica gel chromatography (volume ratio of mobile phase with dichloromethane/methanol = 10:1).

To synthesize inflammatory‐sensitive crosslinkers with disulfide (PEG‐Linker), DMA‐PEG‐DMA (100 mg) was dissolved in extra dry DMF, followed by the addition of DSC (50 mg) and DMAP (5 mg). The mixture was stirred for 1–2 h and then suspended to obtain the crude product. Finally, the product was purified using silica gel chromatography (volume ratio of mobile phase with dichloromethane/isopropyl alcohol = 2:1). ^1^H NMR spectra of macromolecules were recorded on a Bruker 400 MHz nuclear magnetic resonance instrument (Ettlingen, Germany) using deuterated DMSO as the solvents.

### Preparation and Characterization of RVG‐IL3 NGs

PEG‐Linker was dissolved in DMSO at a concentration of 10 mg mL^−1^ and subsequently added to IL‐3 in Dulbecco's phosphate buffer (DPBS) with a molar ratio of 15:1 (PEG‐Linker/IL3). The mixture was gently rotated at a temperature of 25 °C for 30 min. Following this, RVG in DPBS was added to the diluted solution with a molar ratio of 1:10 (RVG/IL3). The mixture was rotated at 25 °C for another 30 min. The resulting RVG‐IL3 NGs were subjected to three washes using 1.5 mL of DPBS through the Amicon ultra centrifugal filter with a molecular weight cutoff of 100 kDa.

The size, size distribution, and zeta potential of IL‐3, IL3 NGs, and RVG‐IL3 NGs were determined using DLS (Zetasizer Nano ZS90, Great Malvern, UK). To study the size variation of RVG‐IL3 NGs in a mildly acidic environment, RVG‐IL3 NGs were dispersed in PBS at a concentration of 0.2 mg mL^−1^ and incubated at 4 °C under either pH 6.5 or 7.4. After a 4 h incubation period, size changes were monitored using DLS. The morphology and structure of RVG‐IL3 NGs were examined using a TEM (FEI Tecnai G2 F20 S‐Twin, Hillsboro, OR).

### In Vivo RVG‐IL3 NGs Targeted Accumulation to PD Brain

To investigate the targeted accumulation of RVG‐IL3 NGs in the PD brain, Cy5‐labeled IL‐3 and RVG‐IL3 NGs (100 µg kg^−1^) were intravenously injected into PD mice. The near‐infrared fluorescence intensity of Cy5 was monitored at various time intervals using a Xenogen IVIS imaging system (Caliper Life Sciences, USA). Mice were euthanized at 24 h post‐injection, and the major organs (heart, liver, spleen, lung, kidney, and brain) were collected for IVIS imaging.

### Open Field Test

An open field test was performed according to a previous work.^[^ ^32^, ^33^ ^]^ In brief, mice were placed in a rectangular plastic box (40 × 40 × 40 cm) and were allowed to move freely for 15 min. The total distance traveled and movement speed were recorded using a video tracking system (EthoVisione XT software, Beijing, China).

### Pole‐Climbing Test

As described previously,^[^ ^32^ ^]^ mice were placed on the top of a pole (length of 75 cm and width of 9 mm), and the time mice took to reach the ground from the top was recorded.

### Grasping Test

As reported previously,^[^ ^32^ ^]^ mice were suspended on a horizontal metal wire (l mm diameter and 30 cm above the ground) for 10 s using the two front paws. The grasping score was recorded as 3, 2, 1, and 0 if mice grasped the wire with two hind paws, mice grasped the wire with one hind paw, mice failed to grasp the wire, or mice fell, respectively.

### Rotarod Test

A rotarod test was performed identically to a previous work.^[^ ^7^, ^32^ ^]^ Mice received training on the rotarod (Ugo Basile SRL, Gemonio, VA, Italy) at a speed of 10 rpm for 3 d. On the test day, mice were placed on the rotarod cylinder, which was accelerated from 4 to 40 rpm within 5 min. The latency time the mice took to fall on the rotarod was recorded.

### Western Blotting

Western blotting was performed according to methods described in a previous study.^[^ ^30^ ^]^ Total proteins were extracted from the SN, striatum, microglia, and MN9D cells with RIPA lysis buffer (Beyotime Biotechnology, Shanghai, China). Protein concentration was quantified using a BCA protein assay kit (Thermo Fisher Scientific, Lenexa, KS). Equal amounts of proteins were resolved on SDS‐PAGE gels followed by electrophoretic transfer onto polyvinylidene fluoride membranes and immunoblotting with the indicated antibodies. Images were captured using a GeneGnome XRQ Chemiluminescence imaging system (Gene Company, Hong Kong, China). Protein bands were quantified using ImageJ software.

### Quantitative Reverse Transcription Polymerase Chain Reaction (qRT‐PCR)

A qRT‐PCR was performed according to methods described in a previous work.^[^ ^7^ ^]^ Total RNA was isolated from the SN, microglia, and MN9D cells using Trizol reagent (Invitrogen, Carlsbad, CA). Then, cDNA synthesis was performed using a cDNA Reverse Transcription Kit (QIAGEN, Waltham, MA). All qPCR assays were performed using SYBR Green PCR Master Mix (Takara, Otsu, Japan). Table S1 (Supporting Information) lists the sequences of qPCR primers. Relative expression was calculated using the ΔΔCt method.

### Migration Assay

Migration assay was performed according to a previous study.^[^ ^34^ ^]^ The assay was conducted in Transwell chambers (8 µm pores), and matrigel was coated on the apical chamber of each Transwell and incubated at 37 °C for 30 min. Then, microglia were seeded on the apical chamber containing 100 µL of serum‐free DMEM, whereas the basolateral chamber was filled with 800 µL of DMEM containing 10% FCS. Plates were incubated at 37 °C in 5% CO_2_ for 24 h. After migration, cells were fixed with 4% paraformaldehyde (PFA) for 30 min and stained with 0.1% crystal violet at room temperature for 30 min. Nonmigrated cells on the top of the membrane matrix were removed using cotton swabs. Migrated cells were observed and quantified using ImageJ software.

### Immunofluorescence Assay

The immunofluorescence assay was performed as stated previously.^[^ ^30^ ^]^ Brains were removed and fixed in PFA solution at 4 °C overnight. Fixed brains were dehydrated in 30% sucrose/0.1 m PBS for 3 d. The serial frozen sections were permeabilized in methanal at −20 °C for 10 min. After the sections were blocked with 5% bovine serum albumin, primary antibodies were incubated at 4 °C overnight. After incubation with a fluorescent‐labeled secondary antibody, images were acquired using a confocal microscope (SP8; Leica). Quantitative analysis was performed using the Image‐Pro Plus 6.0 photogram analysis system (IPP 6.0, Media Cybernetics, Bethesda, MD).

### Transmission Electron Microscopy

TEM was performed according to a previous work.^[^ ^32^ ^]^ SN tissues were quickly placed in an electron microscopy fixation solution and fixed at 4 °C for 2–4 h. After being rinsed with 0.1 m PBS (pH 7.4) for three times, the samples were post‐fixed in 1% osmium tetroxide in 0.1 m PBS (pH 7.4) for 2 h. The samples were then dehydrated with different concentrations of ethanol and acetone. After infiltration overnight, the samples were embedded in resin (Sigma, St. Louis, MO). The embedded samples were sectioned using a Leica ultramicrotome with a thickness of 60–80 nm. After the uranium‐lead double staining, the images of lysosomes were captured using transmission electron microscopy (HT7700; Hitachi, Tokyo, Japan).

### RNA‐seq and Bioinformatic Analysis

SN tissue was isolated, and bulk RNA‐seq was performed, as described previously.^[^ ^32^ ^]^ In brief, RNA was isolated using Trizol (Invitrogen, Carlsbad, CA) according to the manufacturer's instructions. Then, cDNA libraries were prepared using the NEBNext UltraTM RNA Library Prep Kit for Illumina (NEB, USA). Libraries were sequenced on a HiSeq 2500 instrument (Illumina) at the MGH Next Generation Sequencing Core Facility, using paired‐end 50‐bp sequencing. Sequencing reads were mapped by Novogene (Beijing, China). Bioinformatic analysis was performed using the DESeq2 R package (1.10.1). Resulting differential expressed genes (DEGs) were defined by an adjusted *p*‐value  of <0.05 and absolute values of Log2 (fold change) > 0. Kyoto Encyclopedia of Genes and Genomes (KEGG) and Gene Ontology (GO) pathways were used to analyze the enrichment of up‐ or downregulated DEGs.

### Enzyme‐Linked Immunosorbent Assay

The SN tissue was collected, and the protein levels of IL‐1β, IL‐6, TNF‐α, and IL‐10 were examined using ELISA kits according to a previous protocol.^[^ ^30^ ^]^ The optical density values were determined using a Multiscan Spectrum (BioTek, Winooski, VT) at 450 nm, and the results are expressed as pg mL^−1^.

### Statistical Analysis

Data are presented as the mean ± standard error of the mean (SEM). Data were analyzed using a Student's *t*‐test or a one‐way ANOVA, followed by Tukey's post hoc test, as appropriate. Differences with a *p*‐value of <0.05 were considered statistically significant. Statistical analyses were performed using GraphPad Prism 9.0 (GraphPad Software, La Jolla, CA). *P*‐values are represented as ^*^ *p* < 0.05 and ^**^ *p *< 0.01.

## Conflict of Interest

The authors declare no conflict of interest.

## Author Contributions

W.L.Z., J.R., L.Y.D., and S.H.Z. contributed equally to this work. Y.L.Z. and R.J.L. designed the research. W.L.Z. and M.R.Z. performed western blotting. J.R. prepared and provided the RVG‐IL3 NGs system. L.Y.D. and R.F.M. conducted the immunostaining assays. S.H.Z. and Y.L. injected the AAVs and performed the behavioral tests. Y.L.Z., R.J.L., W.L.Z., and J.R. analyzed the data. Y.L.Z. and R.J.L. wrote the manuscript. All authors have read and commented on the manuscript.

## Supporting information

Supporting Information

## Data Availability

The data that support the findings of this study are available from the corresponding author upon reasonable request.

## References

[1] L. V. Kalia , A. E. Lang , Lancet 2015, 386, 896. 25904081 10.1016/S0140-6736(14)61393-3

[2] M. G. Tansey , R. L. Wallings , M. C. Houser , M. K. Herrick , C. E. Keating , V. Joers , Nat. Rev. Immunol. 2022, 22, 657. 35246670 10.1038/s41577-022-00684-6 PMC8895080

[3] a) A. Nimmerjahn , F. Kirchhoff , F. Helmchen , Science 2005, 308, 1314; 15831717 10.1126/science.1110647

[4] S. A. Liddelow , K. A. Guttenplan , L. E. Clarke , F. C. Bennett , C. J. Bohlen , L. Schirmer , M. L. Bennett , A. E. Munch , W. S. Chung , T. C. Peterson , D. K. Wilton , A. Frouin , B. A. Napier , N. Panicker , M. Kumar , M. S. Buckwalter , D. H. Rowitch , V. L. Dawson , T. M. Dawson , B. Stevens , B. A. Barres , Nature 2017, 541, 481. 28099414 10.1038/nature21029 PMC5404890

[5] S. P. Yun , T. I. Kam , N. Panicker , S. Kim , Y. Oh , J. S. Park , S. H. Kwon , Y. J. Park , S. S. Karuppagounder , H. Park , S. Kim , N. Oh , N. A. Kim , S. Lee , S. Brahmachari , X. Mao , J. H. Lee , M. Kumar , D. An , S. U. Kang , Y. Lee , K. C. Lee , D. H. Na , D. Kim , S. H. Lee , V. V. Roschke , S. A. Liddelow , Z. Mari , B. A. Barres , V. L. Dawson , et al., Nat. Med. 2018, 24, 931. 29892066 10.1038/s41591-018-0051-5 PMC6039259

[6] a) T. Zhou , Y. Li , X. Li , F. Zeng , Y. Rao , Y. He , Y. Wang , M. Liu , D. Li , Z. Xu , X. Zhou , S. Du , F. Niu , J. Peng , X. Mei , S. J. Ji , Y. Shu , W. Lu , F. Guo , T. Wu , T. F. Yuan , Y. Mao , B. Peng , Nat. Commun. 2022, 13, 6233; 36280666 10.1038/s41467-022-33932-3 PMC9592609

[7] W. Zhang , L. Ding , H. Chen , M. Zhang , R. Ma , S. Zheng , J. Gong , Z. Zhang , H. Xu , P. Xu , Y. Zhang , Cell Death Dis. 2023, 14, 285. 37087484 10.1038/s41419-023-05807-y PMC10122675

[8] C. S. McAlpine , J. Park , A. Griciuc , E. Kim , S. H. Choi , Y. Iwamoto , M. G. Kiss , K. A. Christie , C. Vinegoni , W. C. Poller , J. E. Mindur , C. T. Chan , S. He , H. Janssen , L. P. Wong , J. Downey , S. Singh , A. Anzai , F. Kahles , M. Jorfi , P. F. Feruglio , R. I. Sadreyev , R. Weissleder , B. P. Kleinstiver , M. Nahrendorf , R. E. Tanzi , F. K. Swirski , Nature 2021, 595, 701. 34262178 10.1038/s41586-021-03734-6 PMC8934148

[9] L. Basurco , M. A. Abellanas , L. Ayerra , E. Conde , R. Vinueza‐Gavilanes , E. Luquin , A. Vales , A. Vilas , P. S. Martin‐Uriz , I. Tamayo , M. M. Alonso , M. Hernaez , G. Gonzalez‐Aseguinolaza , P. Clavero , E. Mengual , M. Arrasate , S. Hervas‐Stubbs , M. S. Aymerich , Glia 2023, 71, 571. 36353934 10.1002/glia.24295 PMC10100513

[10] a) M. Dougan , G. Dranoff , S. K. Dougan , Immunity 2019, 50, 796; 30995500 10.1016/j.immuni.2019.03.022 PMC12512237

[11] M. G. Kiss , J. E. Mindur , A. G. Yates , D. Lee , J. F. Fullard , A. Anzai , W. C. Poller , K. A. Christie , Y. Iwamoto , V. Roudko , J. Downey , C. T. Chan , P. Huynh , H. Janssen , A. Ntranos , J. D. Hoffmann , W. Jacob , S. Goswami , S. Singh , D. Leppert , J. Kuhle , S. Kim‐Schulze , M. Nahrendorf , B. P. Kleinstiver , F. Probert , P. Roussos , F. K. Swirski , C. S. McAlpine , Immunity 2023, 56, 1502. 37160117 10.1016/j.immuni.2023.04.013 PMC10524830

[12] S. Mitragotri , P. A. Burke , R. Langer , Nat. Rev. Drug Discovery 2014, 13, 655. 25103255 10.1038/nrd4363 PMC4455970

[13] a) C. Chittasupho , S. Tadtong , S. Vorarat , W. Imaram , S. Athikomkulchai , W. Samee , V. Sareedenchai , T. Thongnopkoon , S. Okonogi , N. Kamkaen , Pharmaceutics 2022, 14, 1079; 35631666 10.3390/pharmaceutics14051079 PMC9147856

[14] a) S. Boridy , H. Takahashi , K. Akiyoshi , D. Maysinger , Biomaterials 2009, 30, 5583; 19577802 10.1016/j.biomaterials.2009.06.010

[15] N. Ramalingam , U. Dettmer , Mol. Neurodegener. 2023, 18, 84. 37953316 10.1186/s13024-023-00680-x PMC10641962

[16] B. Z. Qian , J. Li , H. Zhang , T. Kitamura , J. Zhang , L. R. Campion , E. A. Kaiser , L. A. Snyder , J. W. Pollard , Nature 2011, 475, 222. 21654748 10.1038/nature10138 PMC3208506

[17] P. Ejlerskov , J. G. Hultberg , J. Wang , R. Carlsson , M. Ambjorn , M. Kuss , Y. Liu , G. Porcu , K. Kolkova , C. Friis Rundsten , K. Ruscher , B. Pakkenberg , T. Goldmann , D. Loreth , M. Prinz , D. C. Rubinsztein , S. Issazadeh‐Navikas , Cell 2015, 163, 324. 26451483 10.1016/j.cell.2015.08.069 PMC4601085

[18] a) S. He , C. Wang , H. Dong , F. Xia , H. Zhou , X. Jiang , C. Pei , H. Ren , H. Li , R. Li , H. Xu , Autophagy 2012, 8, 1621; 22874556 10.4161/auto.21561 PMC3494591

[19] W. Zhang , H. Chen , L. Ding , J. Gong , M. Zhang , W. Guo , P. Xu , S. Li , Y. Zhang , Adv. Sci. 2021, 8, 2004555. 10.1002/advs.202004555 PMC8097374 33977069

[20] Y. Ben‐Shlomo , S. Darweesh , J. Llibre‐Guerra , C. Marras , M. San Luciano , C. Tanner , Lancet 2024, 403, 283. 38245248 10.1016/S0140-6736(23)01419-8 PMC11123577

[21] a) Y. Mi , G. Qi , F. Vitali , Y. Shang , A. C. Raikes , T. Wang , Y. Jin , R. D. Brinton , H. Gu , F. Yin , Nat. Metab. 2023, 5, 445; 36959514 10.1038/s42255-023-00756-4 PMC10202034

[22] L. Yang , C. Wu , E. Parker , Y. Li , Y. Dong , L. Tucker , D. W. Brann , H. W. Lin , Q. Zhang , Theranostics 2022, 12, 2205. 35265207 10.7150/thno.70756 PMC8899582

[23] M. E. Choudhury , K. Sugimoto , M. Kubo , M. Nagai , M. Nomoto , H. Takahashi , H. Yano , J. Tanaka , Brain Behav. 2011, 1, 26. 22398979 10.1002/brb3.11 PMC3217672

[24] T. C. Wen , J. Tanaka , H. Peng , J. Desaki , S. Matsuda , N. Maeda , H. Fujita , K. Sato , M. Sakanaka , J. Exp. Med. 1998, 188, 635. 9705946 10.1084/jem.188.4.635 PMC2213360

[25] a) I. Choi , Y. Zhang , S. P. Seegobin , M. Pruvost , Q. Wang , K. Purtell , B. Zhang , Z. Yue , Nat. Commun. 2020, 11, 1386; 32170061 10.1038/s41467-020-15119-w PMC7069981

[26] a) Y. Rong , C. Ji , Z. Wang , X. Ge , J. Wang , W. Ye , P. Tang , D. Jiang , J. Fan , G. Yin , W. Liu , W. Cai , J. Neuroinflammation 2021, 18, 196; 34511129 10.1186/s12974-021-02268-y PMC8436564

[27] a) Y. Konishi , M. Kamegai , K. Takahashi , T. Kunishita , T. Tabira , Neurosci. Lett. 1994, 182, 271; 7715825 10.1016/0304-3940(94)90814-1

[28] C. Nehammer , P. Ejlerskov , S. Gopal , A. Handley , L. Ng , P. Moreira , H. Lee , S. Issazadeh‐Navikas , D. C. Rubinsztein , R. Pocock , Elife 2019, 8, e49930. 31799933 10.7554/eLife.49930 PMC6914338

[29] T. Nishihara , M. Ochi , K. Sugimoto , H. Takahashi , H. Yano , Y. Kumon , T. Ohnishi , J. Tanaka , Exp. Neurol. 2011, 229, 507. 21515263 10.1016/j.expneurol.2011.04.006

[30] M. Zhang , H. Chen , W. Zhang , Y. Liu , L. Ding , J. Gong , R. Ma , S. Zheng , Y. Zhang , Adv. Sci. 2023, 10, e2300180. 10.1002/advs.202300180 PMC10131853 36799538

[31] Y. Liu , J. Ren , W. Zhang , L. Ding , R. Ma , M. Zhang , S. Zheng , R. Liang , Y. Zhang , Biomaterials 2024, 312, 122707. 39121729 10.1016/j.biomaterials.2024.122707

[32] W. Zhang , L. Ding , M. Zhang , S. Zheng , R. Ma , J. Gong , H. Mao , H. Xu , P. Xu , Y. Zhang , Cell. Mol. Life Sci. 2023, 80, 155. 37204481 10.1007/s00018-023-04807-7 PMC11073026

[33] W. Zhang , H. Chen , L. Ding , J. Huang , M. Zhang , Y. Liu , R. Ma , S. Zheng , J. Gong , J. C. Pina‐Crespo , Y. Zhang , Exploration 2023, 3, 20220160. 37933376 10.1002/EXP.20220160 PMC10624376

[34] J. Lee , K. Pang , J. Kim , E. Hong , J. Lee , H. J. Cho , J. Park , M. Son , S. Park , M. Lee , A. Ooshima , K. S. Park , H. K. Yang , K. M. Yang , S. J. Kim , Nat. Commun. 2022, 13, 6274. 36307405 10.1038/s41467-022-33786-9 PMC9616898

