# Supplementary material for: Nanotherapeutic Approaches of Interleukin‐3 to Clear the α‐Synuclein Pathology in Mouse Models of Parkinson's Disease
Source: Adv Sci (Weinh). 2024 Sep 3;11(42):2405364. doi: 10.1002/advs.202405364 (PMC11558132; doi:10.1002/advs.202405364)
Supplement: Supplementary file 1 — Supporting Information [file ADVS-11-2405364-s001.docx]

**Supporting Information**

**
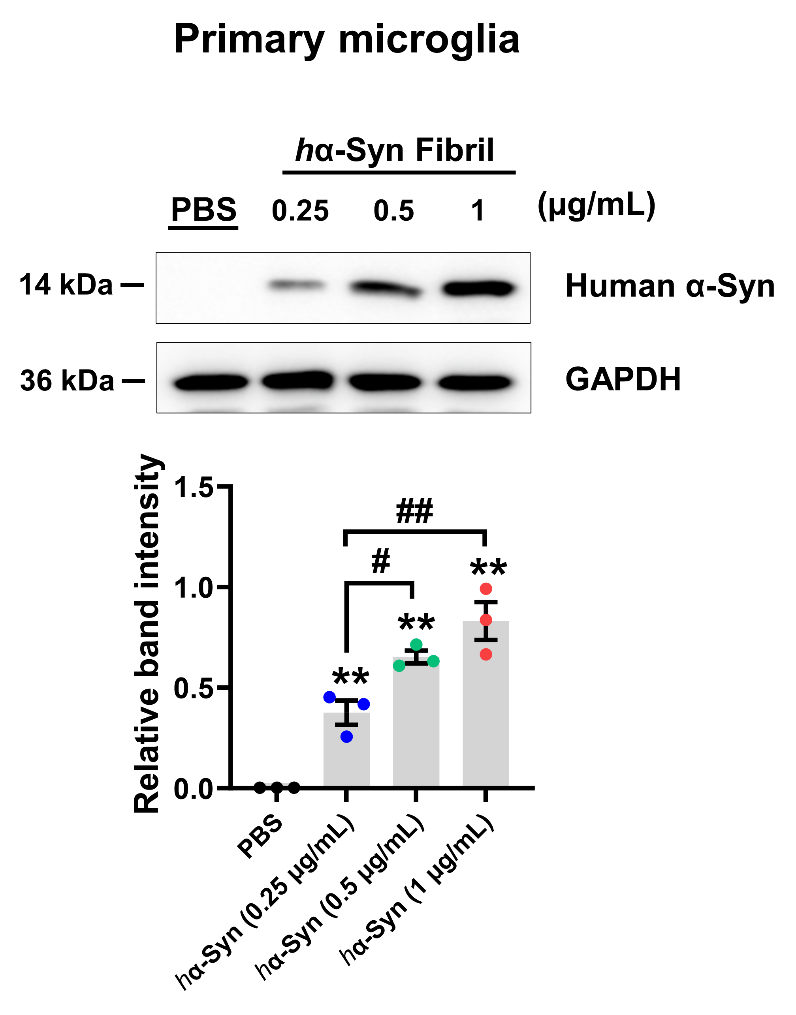
**

**Supplementary Figure S1. Effects of different concentrations of *h*α-Syn fibril in primary microglia.** Representative blots and quantification showing the expression of human α-synuclein in primary microglia treated with 0.25, 0.5, and 1 μg/mL of *h*α-Syn fibril (*n* = 3 per group). Results are expressed as mean ± SEM. ^**^*p* < 0.01 versus PBS; ^##^*p* < 0.01, ^#^*p* < 0.05 versus *h*α-Syn fibril (0.25 μg/mL). A one-way analysis of variance (ANOVA) and a Tukey’s test for the *post hoc* comparisons were performed to determine the statistical significance.


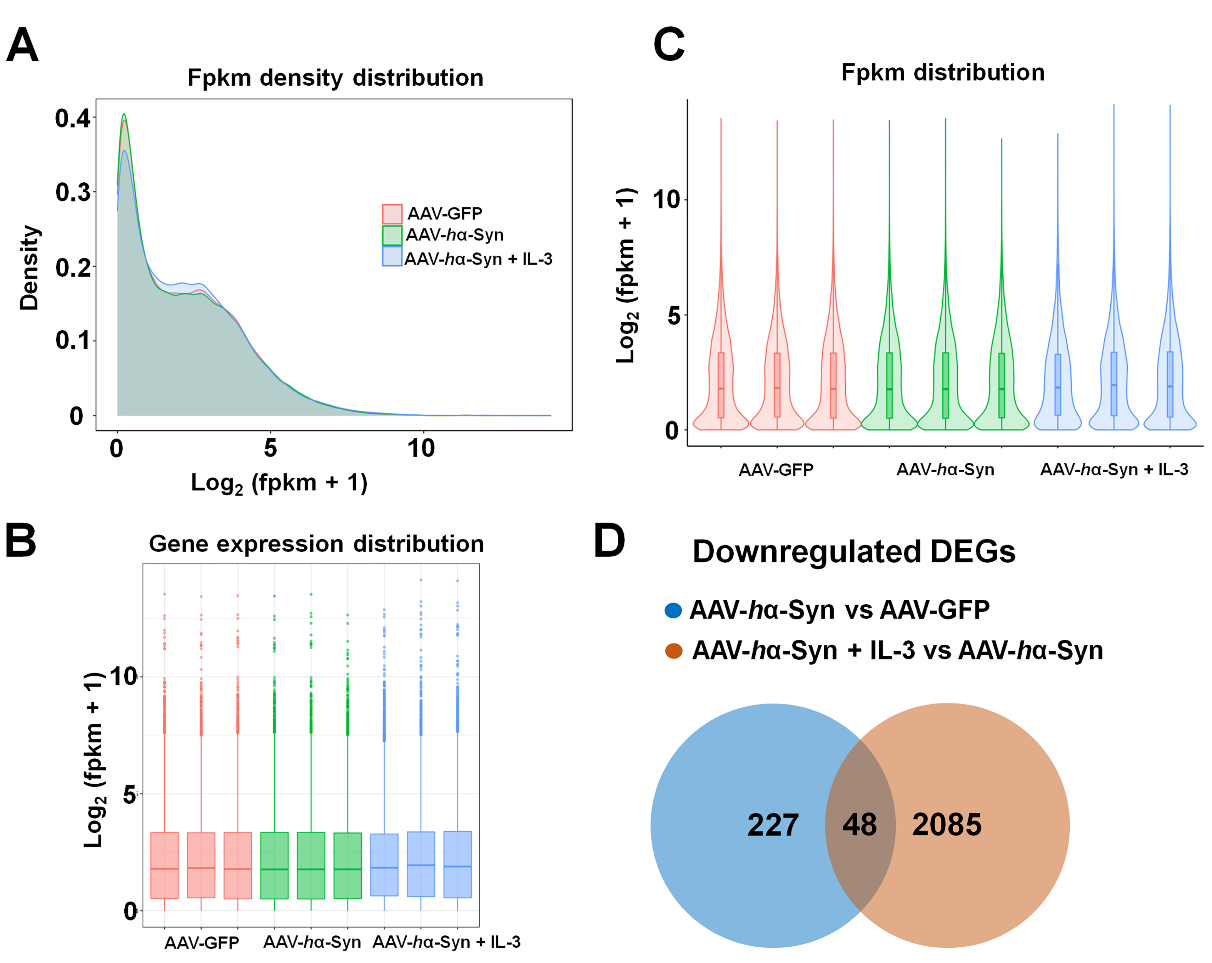


**Supplementary Figure S2. RNA-seq profiles.** (A–C) The Fpkm density distribution, gene expression distribution, and Fpkm distribution in the AAV-GFP, AAV-*h*α-Syn, and AAV-*h*α-Syn + IL-3 mice. (D) Venn diagram showing the number of IL-3 downregulated differential gene expressions (DEGs) in the AAV-*h*α-Syn mice that were increased in the AAV-*h*α-Syn compared with the AAV-GFP mice.


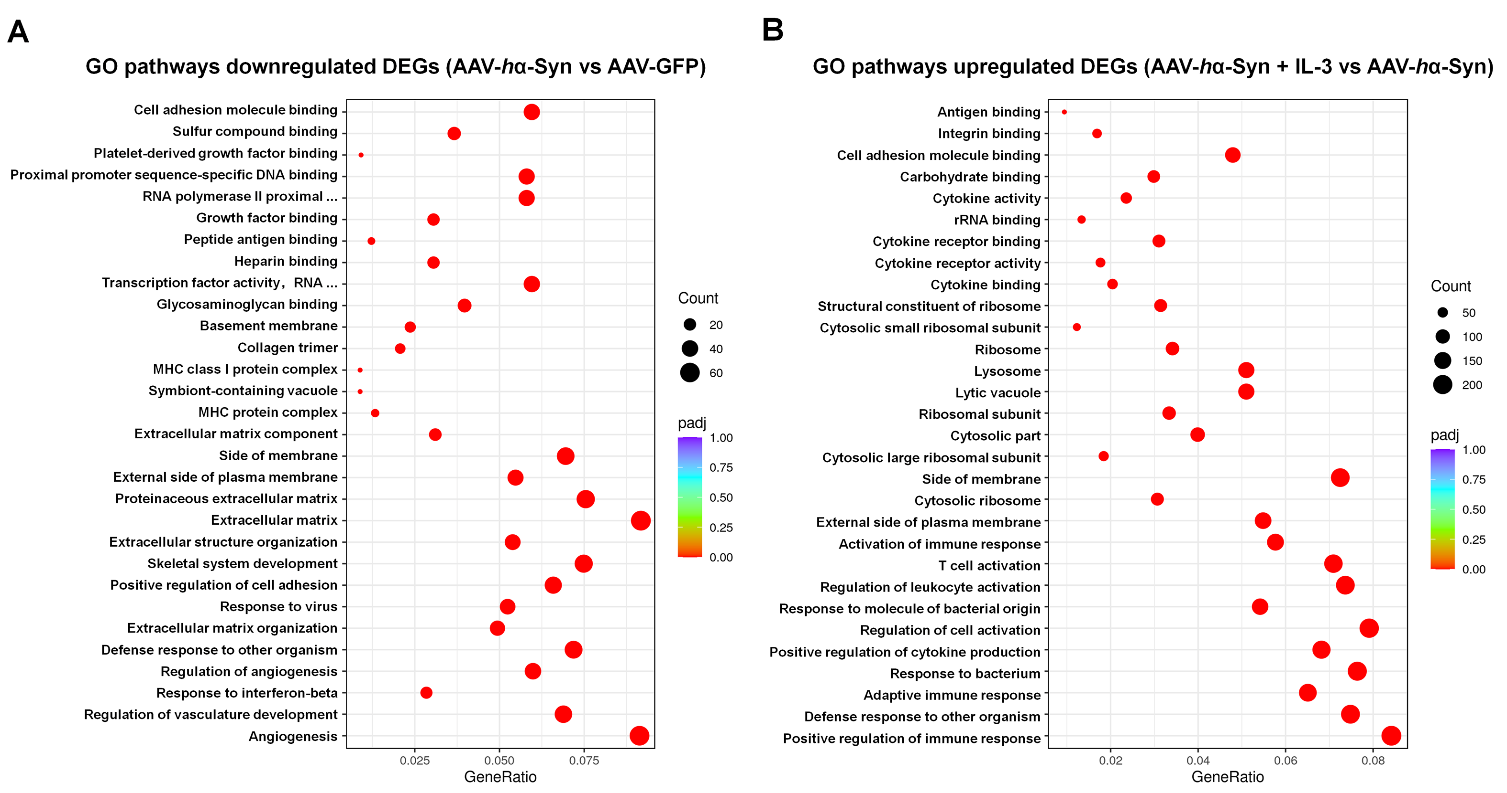


**Supplementary Figure S3. GO pathways enriched by DEGs.** (A) GO pathways enriched by downregulated DEGs (AAV-*h*α-Syn vs. AAV-GFP). (B) GO pathways enriched by upregulated DEGs (AAV-*h*α-Syn + IL-3 vs. AAV-*h*α-Syn).


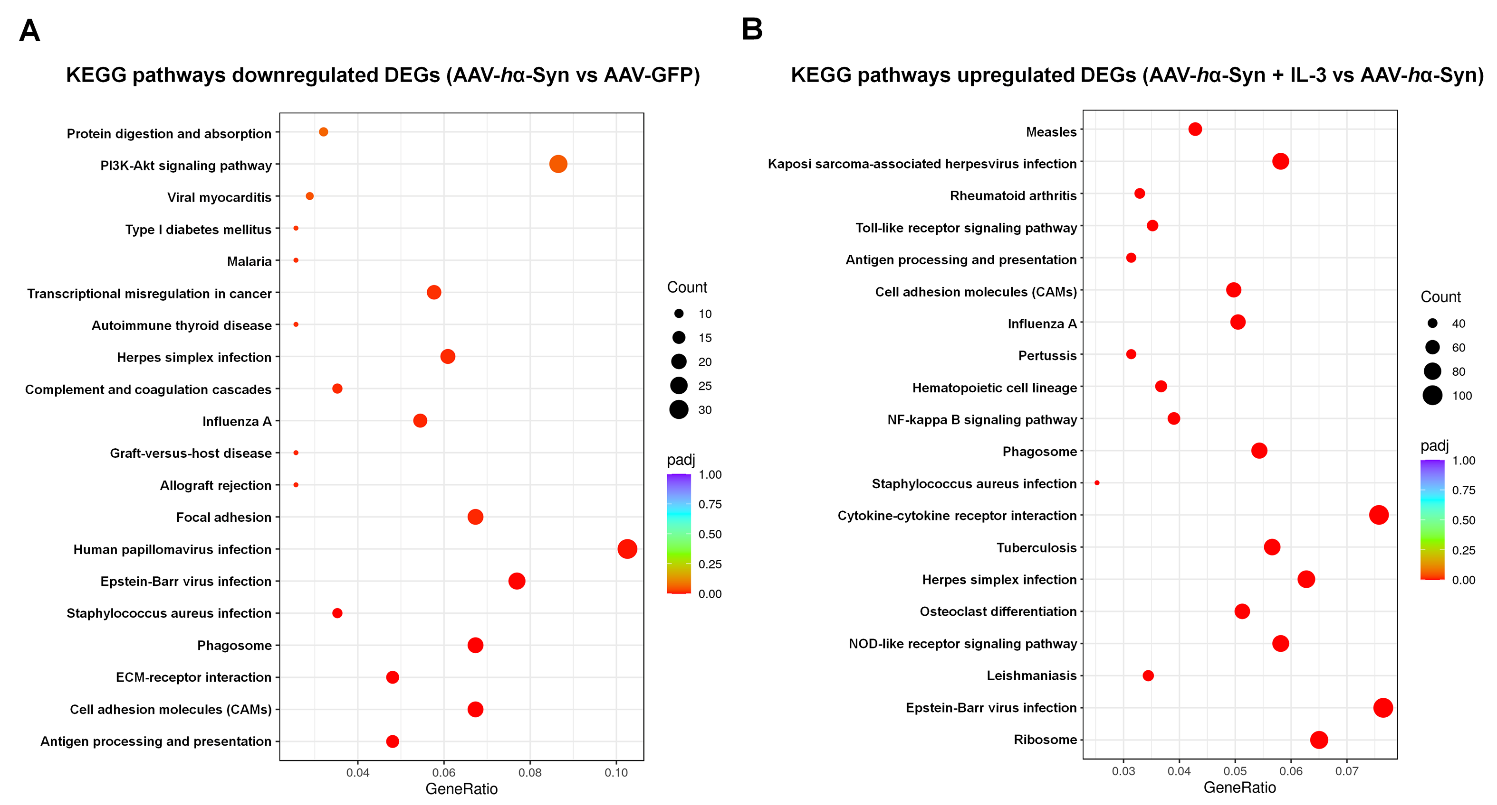


**Supplementary Figure S4. KEGG pathways enriched by DEGs.** (A) KEGG pathways enriched by downregulated DEGs (AAV-*h*α-Syn vs. AAV-GFP). (B) KEGG pathways enriched by upregulated DEGs (AAV-*h*α-Syn + IL-3 vs. AAV-*h*α-Syn).

**
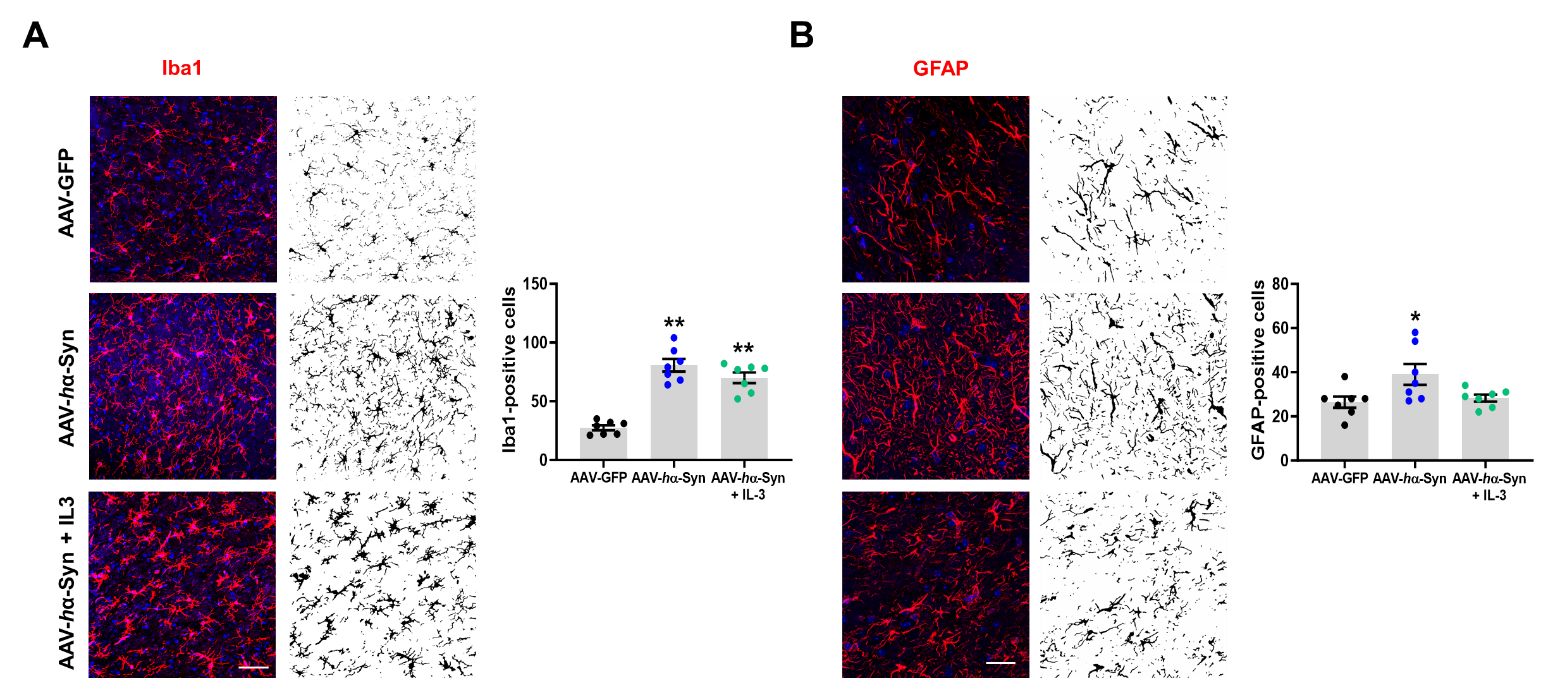
**

**Supplementary Figure S5. Effect of IL-3 on the microglial and astroglial activation in AAV-*h*α-Syn mice.** (A) Immunostaining and quantification showing the number of Iba1-positive cells in the SNpc. *n* = 7 from 3 mice in each group. Scale bar, 50 μm. (G) Immunostaining and quantification showing the number of GFAP-positive cells in the SNpc. *n* = 7 from 3 mice in each group. Scale bar, 25 μm. Results are expressed as mean ± SEM. ^**^*p* < 0.01, ^*^*p* < 0.05 versus AAV-GFP. A one-way ANOVA and a Tukey’s test for *post hoc* comparisons were performed to determine the statistical significance.

**
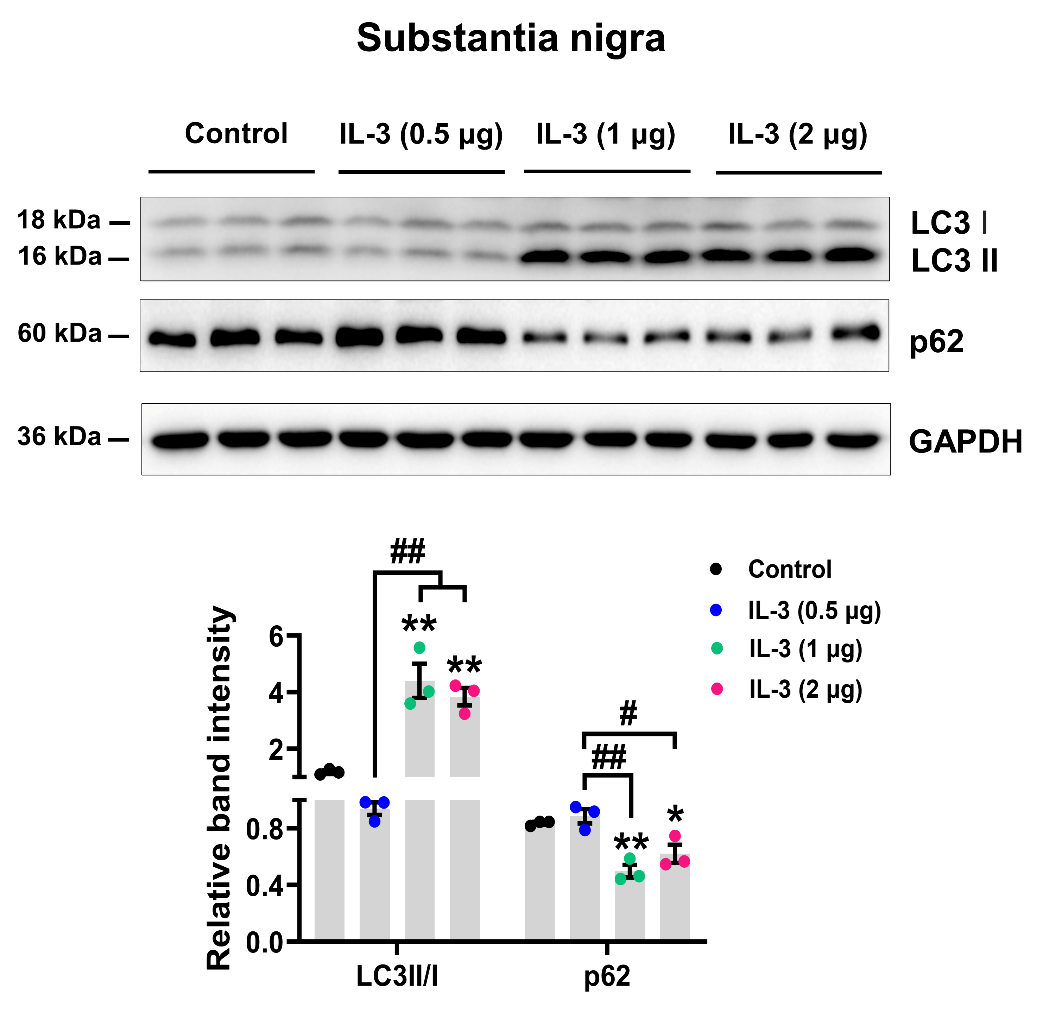
**

**Supplementary Figure S6. Free IL-3 promoted autophagy in the SN of WT mice.** Mice were stereotactically injected with 0.5, 1, and 2 μg free IL-3. After 48 h, the SN was collected for western blotting. Representative blots and quantification showing the expression of LC3 II/I and p62 in the SN of mice treated with PBS, 0.5, 1, and 2 μg of free IL-3. *n* = 3 per group. Results are expressed as mean ± SEM. ^**^*p* < 0.01, ^*^*p* < 0.05 versus Control; ^##^*p* < 0.01, ^#^*p* < 0.05 versus 0.5 μg IL-3. A one-way ANOVA and a Tukey’s test for *post hoc* comparisons were performed to determine the statistical significance.

**
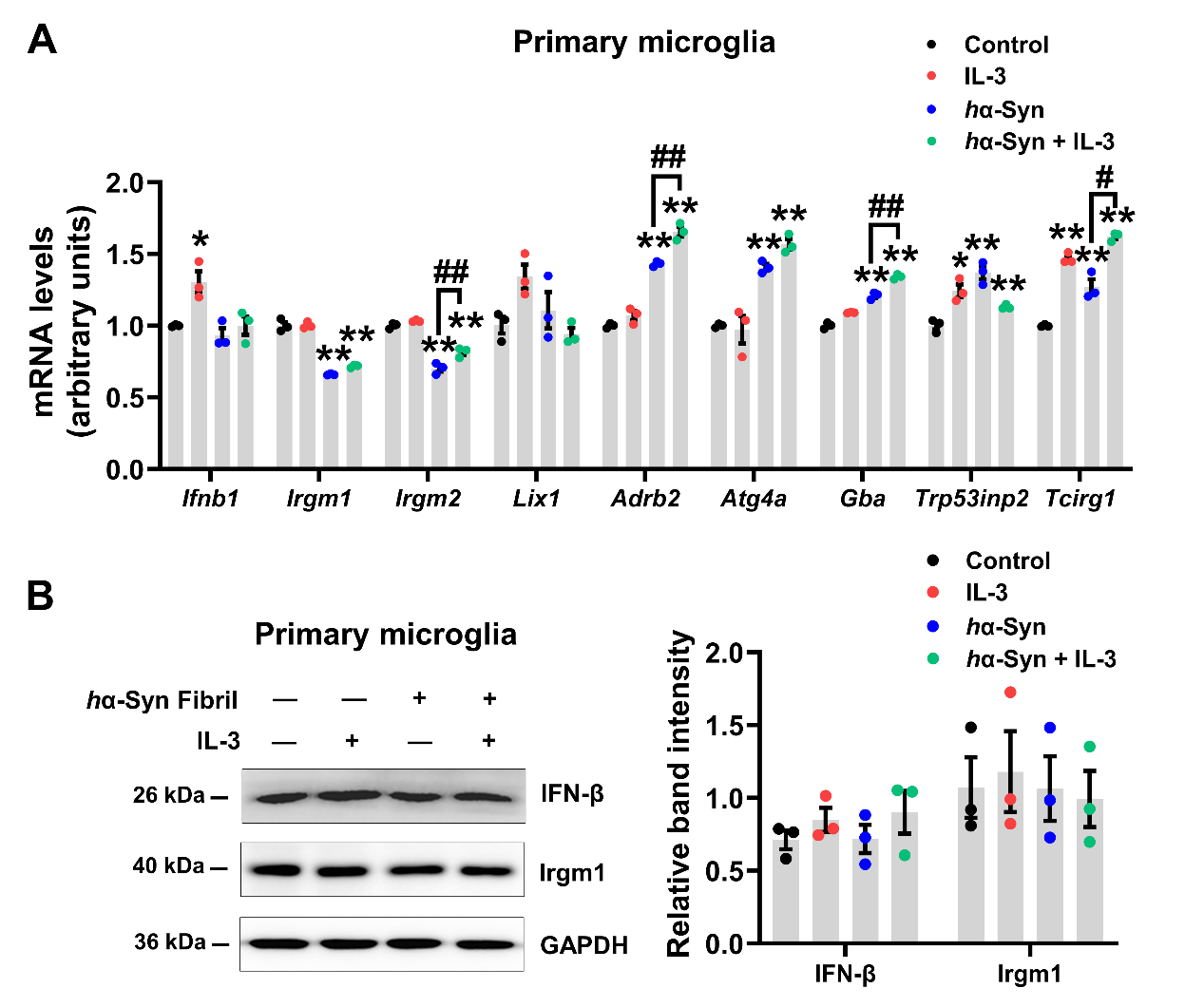
**

**Supplementary Figure S7. Effects of IL-3 on IFN-β/Irgm1 expression in microglia.** (A) mRNA expression levels of *Ifnb1*, *Irgm1*, *Irgm2*, *Lix1*, *Adrb2*, *Atg4a*, *Gba*, *Trp53inp2,* and *Tcirg1* in IL-3 and/or *h*α-Syn fibril-treated primary microglia (*n* = 3 per group). (B) Representative blots and quantification showing the expression of IFN-β and Irgm1 upon IL-3 treatment in *h*α-Syn fibril-treated microglia (*n* = 3 per group). Results are expressed as mean ± SEM. ^**^*p* < 0.01, ^*^*p* < 0.05 versus control; ^##^*p* < 0.01, ^#^*p* < 0.05 versus *h*α-Syn fibril. A one-way ANOVA and a Tukey’s test for *post hoc* comparisons were performed to determine statistical significance.

**
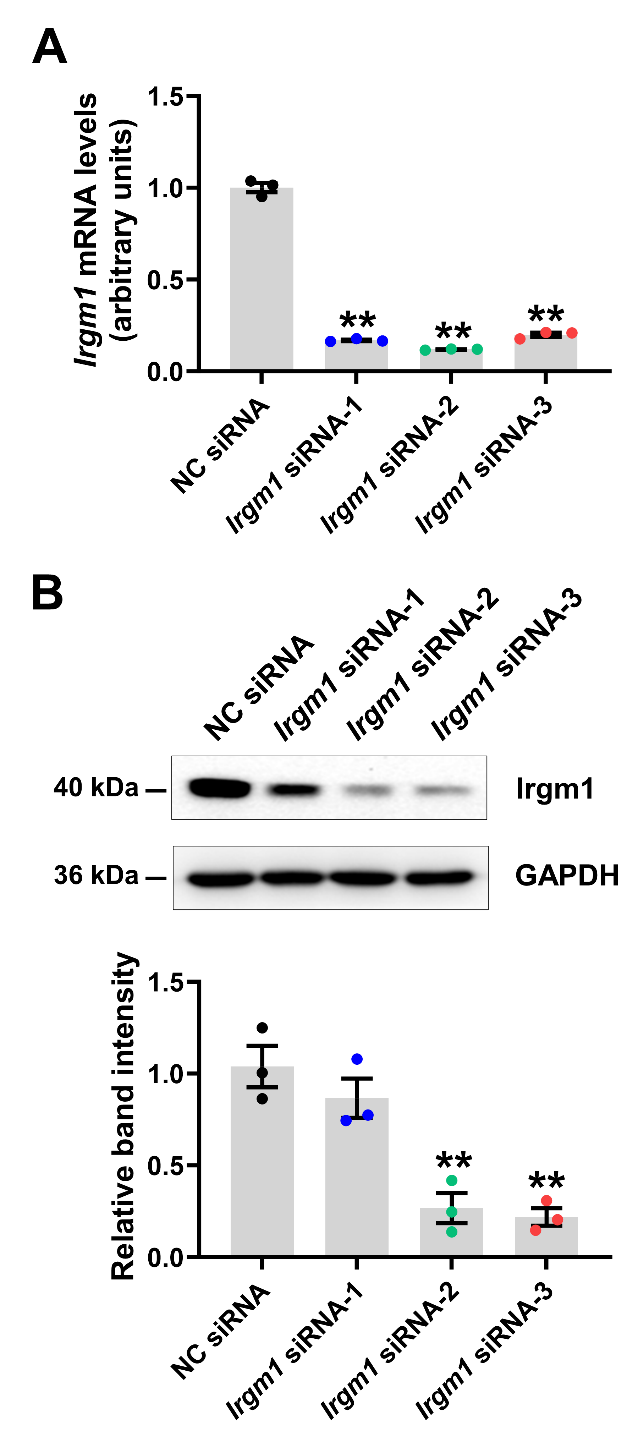
**

**Supplementary Figure S8. Knockdown efficiency of Irgm1 siRNA in MN9D cells.** (A) *Irgm1* mRNA expression level in MN9D cells treated with siRNAs 1–3 (*n* = 3 per group). (B) Representative blots and quantification showing the expression of Irgm1 in MN9D cells treated with siRNAs 1–3 (*n* = 3 per group). Results are expressed as mean ± SEM. ^**^*p* < 0.01 versus NC siRNA. A one-way ANOVA and a Tukey’s test for *post hoc* comparisons were performed to determine statistical significance.

**
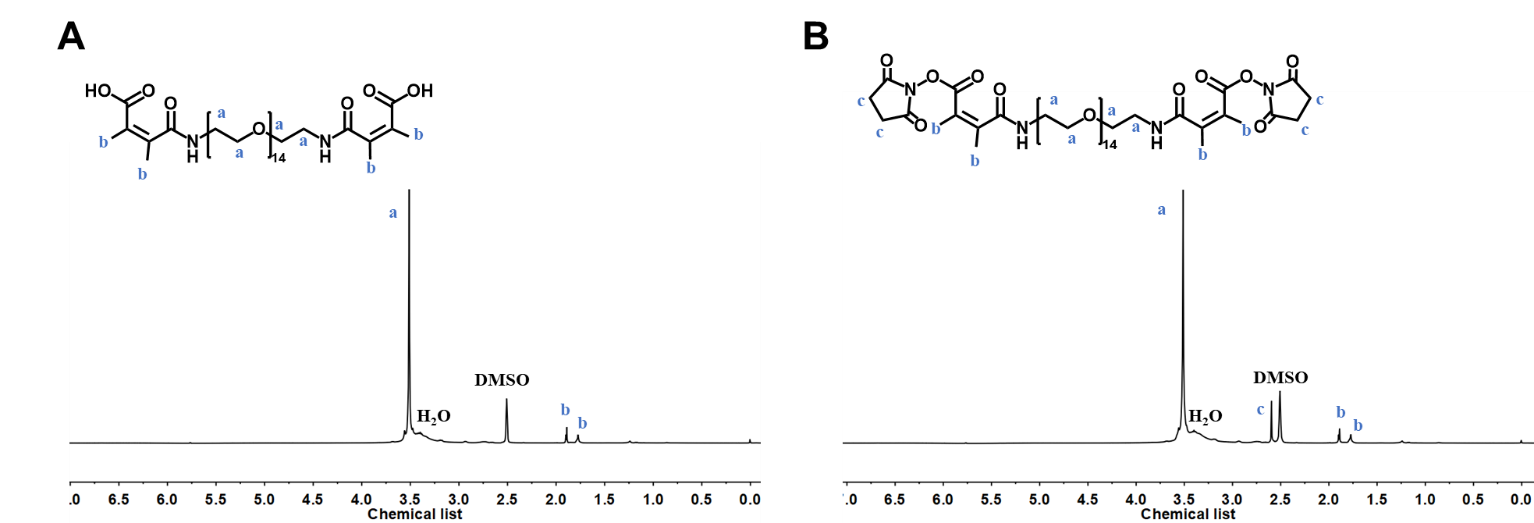
**

**Supplementary Figure S9. ^1^H NMR spectrum of (A) DMA-PEG-DMA and (B) PEG-Linker.**


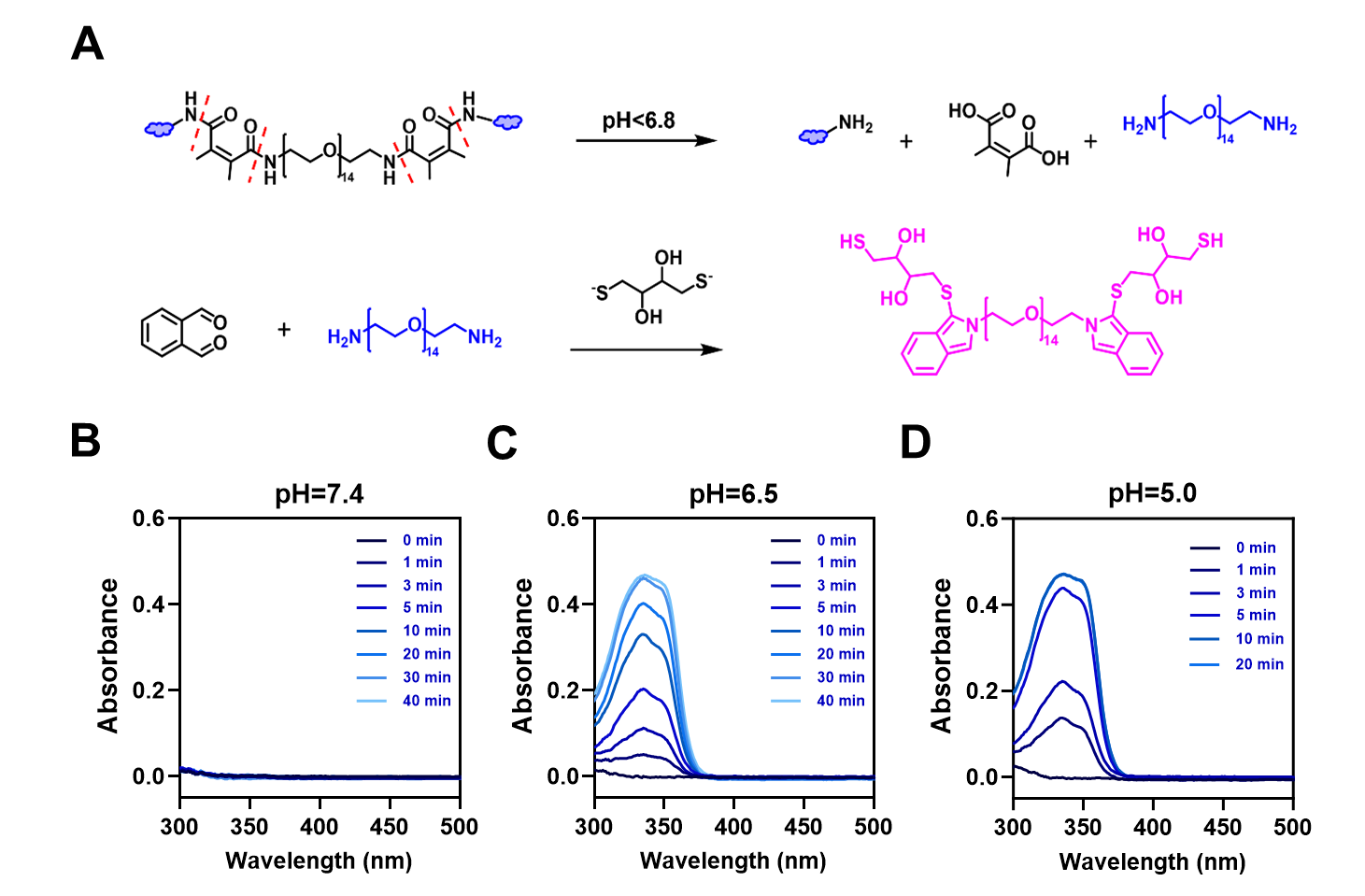


**Supplementary Figure S10. pH-Responsive fracture Study of PEG-Linker using UV spectroscopy.** (A) Reaction steps and mechanism that describe the OPA reaction to detect the exposed primary amines of PEG-Linker. (B-D) UV spectra of the DMA-PEG-DMA dispersed in solutions with varying pH levels and treated with the OPA reagent. The DMA-PEG-DMA (30 mg/mL) were diluted in aqueous solutions with pH values of 7.4, 6.5, and 5.0. After 0, 1, 3, 5, 10, 20, 30, and 40 m, 10 μL of the solution was mixed with 500 μL of the OPA reagent. After two minutes, the absorbance from 300 nm to 500 nm was measured using UV spectroscopy.


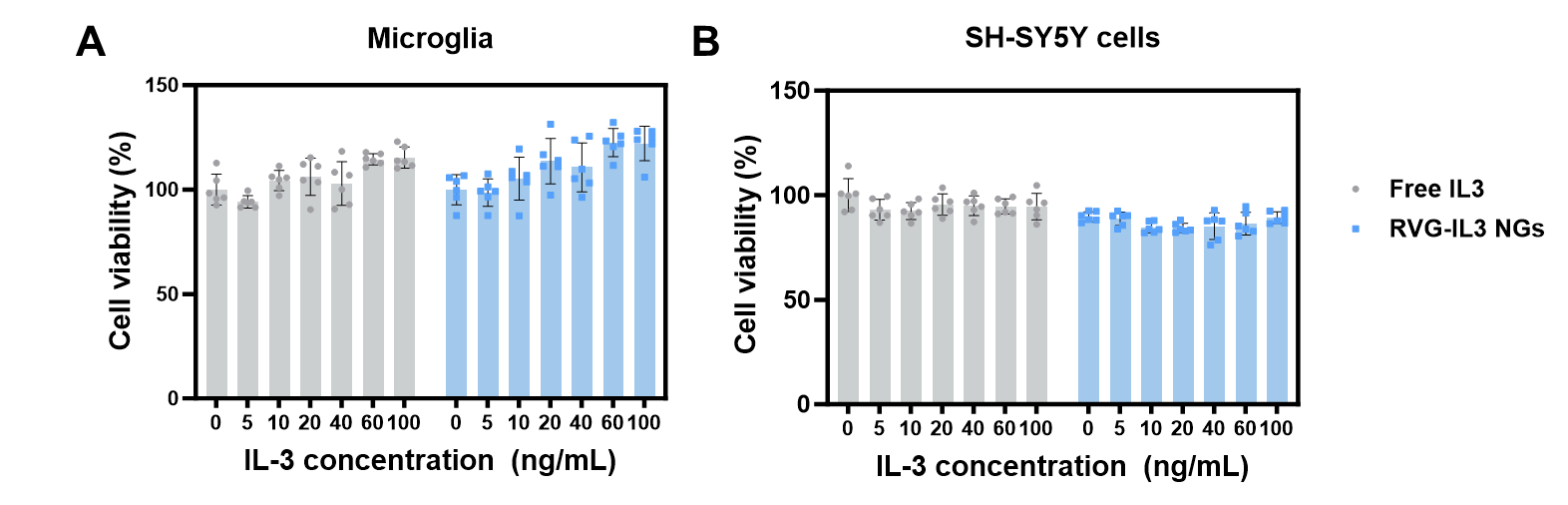


**Supplementary Figure S11.** Cell viability of microglia and the SH-SY5Y cell line treated with free IL-3 and RVG IL-3 NGs via the CCK8 assay. The microglia and SH-SY5Y cells were seeded into 96-well plates (1×10^4^ per well). IL-3 and RVG IL-3 NGs concentrations (0, 5, 10, 20, 40, 60, 100 μg/mL) were incubated with microglia and the SH-SY5Y cells for 48 h. The CCK8 assays were then employed to assess the cell viability.

**
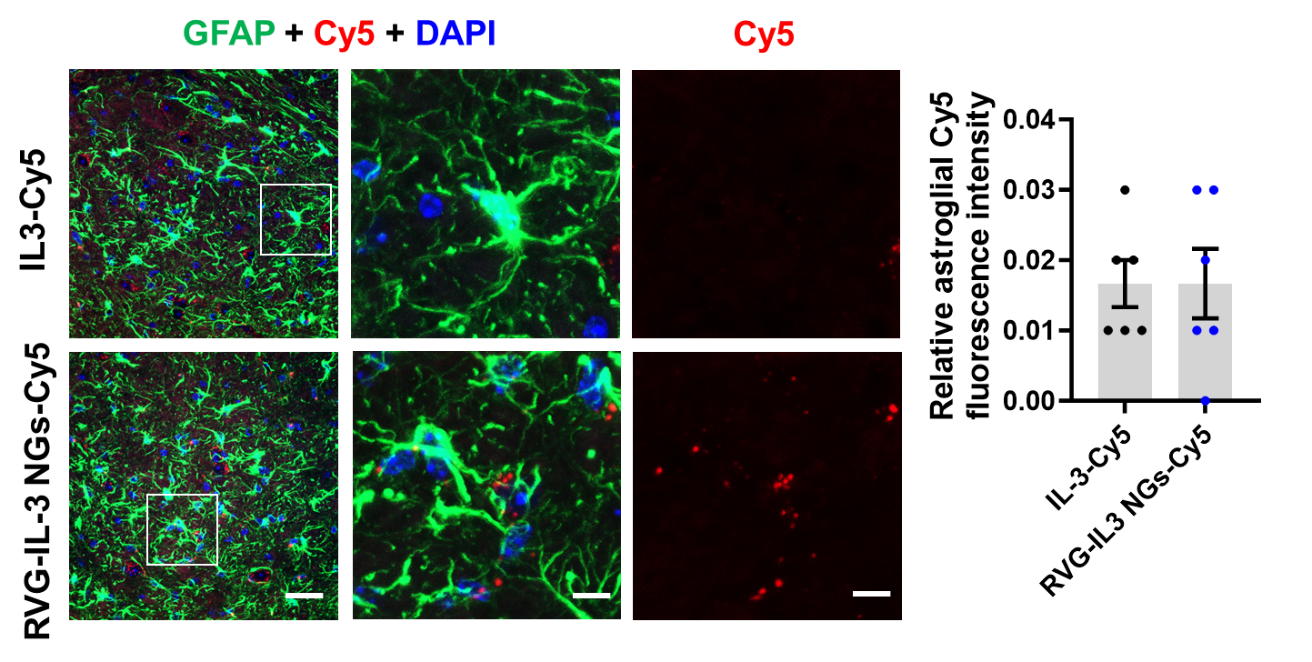
**

**Supplementary Figure S12. Distribution of the Cy5-labeled RVG-IL3 NGs in astrocytes.** Immunostaining of Cy5-labeled IL-3 and Cy5-labeled RVG-IL3 NGs in GFAP-positive cells in the SNpc. Scale bar, 20 μm. Magnified images are shown on the right. Scale bar, 6.5 μm. *n* = 6 per group. Results are expressed as mean ± SEM. A Student’s *t*-test was performed to determine statistical significance.


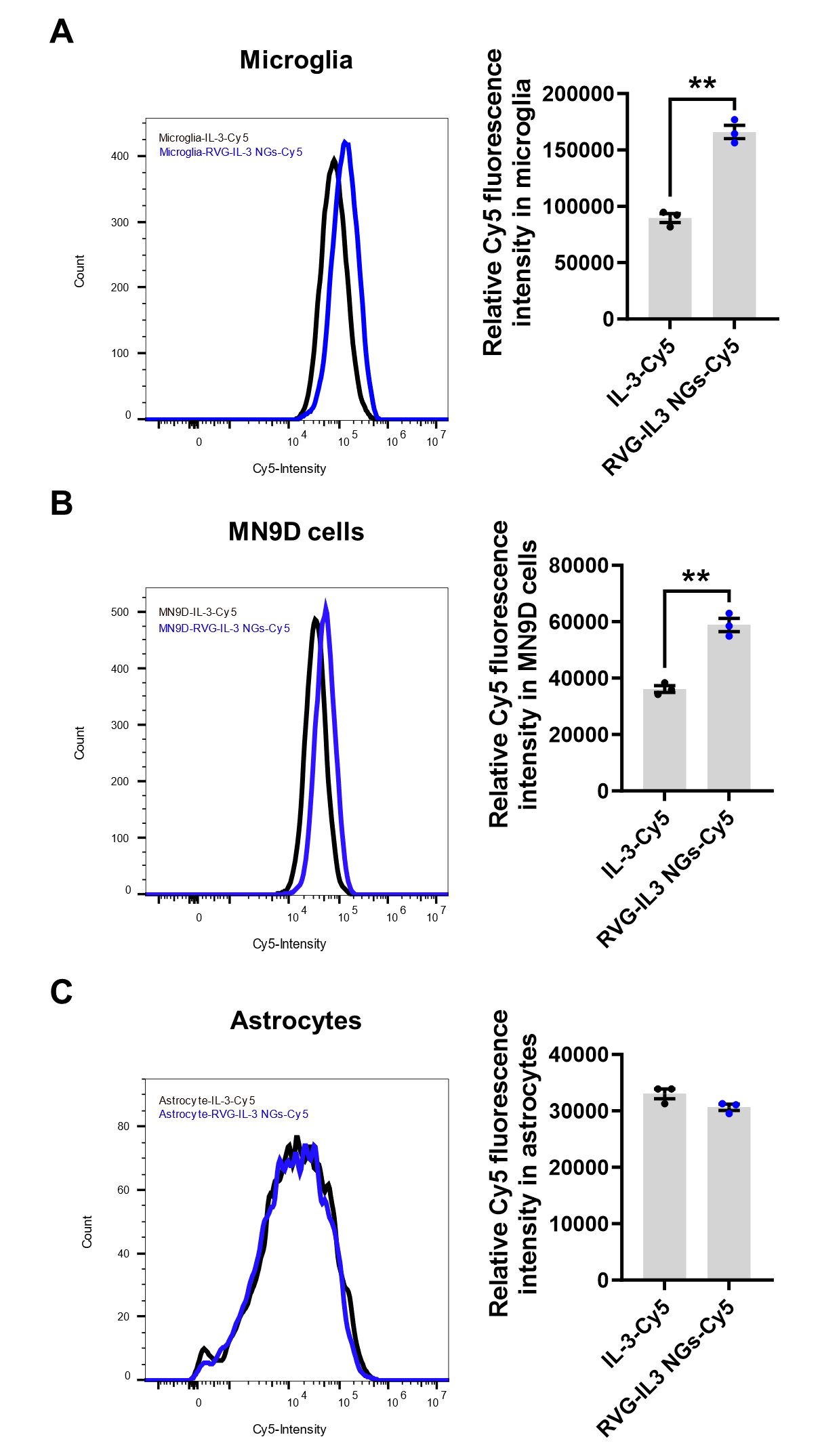


**Supplementary Figure S13. Flow cytometry analysis of cellular uptake of the Cy5-labeled RVG-IL3 NGs.** Flow cytometry analysis and quantification of cellular uptake of the Cy5-labeled IL3 and Cy5-labeled RVG-IL3 NGs in microglia (A), MN9D cells (B), and astrocytes (C). *n* = 3 per group. Results are expressed as mean ± SEM. ^**^*p* < 0.01 versus free IL-3. A student’s *t*-test was performed to determine the statistical significance.

**
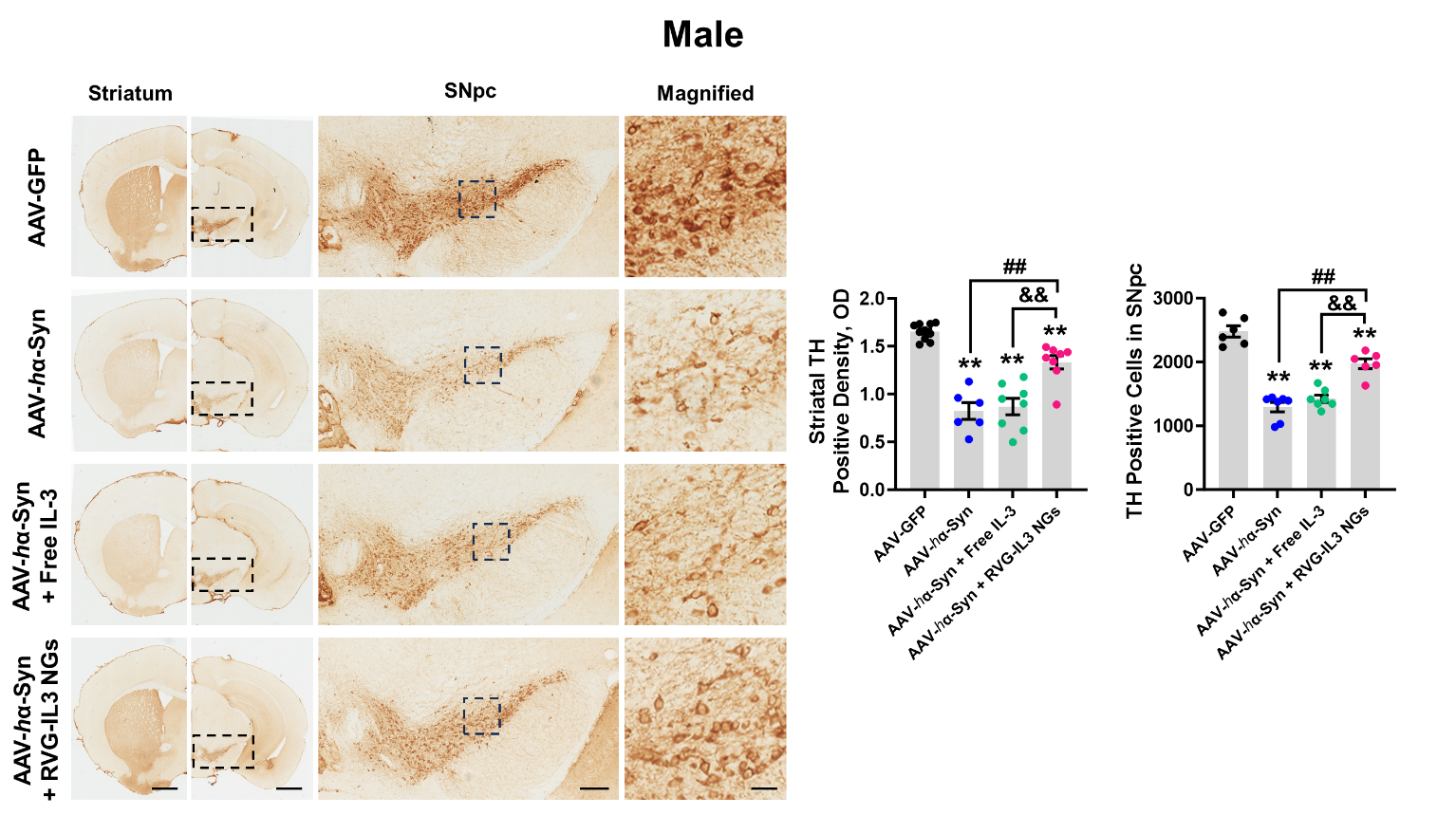
**

**Supplementary Figure S14. Effect of RVG-IL3 NGs on the DA neuronal survival in male AAV-*h*α-Syn mice.** Immunohistochemistry staining and quantification of TH-positive density in the striatum and TH-positive cells in the SNpc of male AAV-GFP, AAV-*h*α-Syn, AAV-*h*α-Syn + free IL-3, and AAV-*h*α-Syn + RVG-IL3 NGs mice. Scale bars, 1 mm for striatum and 500 μm for SNpc. Magnified images of TH-positive cells in the SNpc are shown in the right column (*n* = 6–10 from 3 mice in each group). Scale bars, 50 μm. Results are expressed as mean ± SEM. ^**^*p* < 0.01 versus AAV-GFP; ^##^*p* < 0.01 versus AAV-*h*α-Syn; ^&&^*p* < 0.01 versus AAV-*h*α-Syn + free IL-3. A one-way ANOVA and a Tukey’s test for *post hoc* comparisons were performed to determine statistical significance.

**
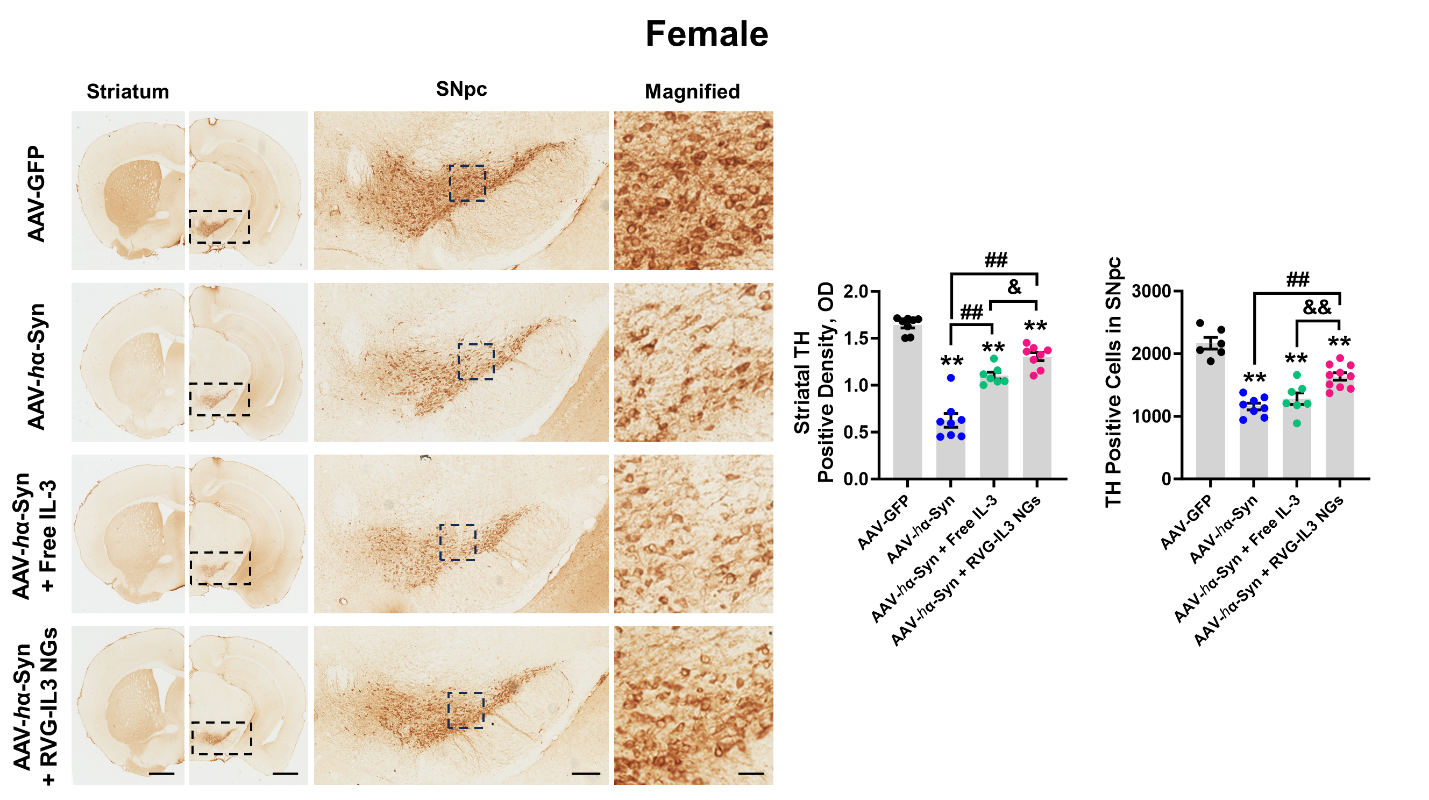
**

**Supplementary Figure S15. Effect of RVG-IL3 NGs on the DA neuronal survival in female AAV-*h*α-Syn mice.** Immunohistochemistry staining and quantification of TH-positive density in the striatum and TH-positive cells in the SNpc of female AAV-GFP, AAV-*h*α-Syn, AAV-*h*α-Syn + free IL-3, and AAV-*h*α-Syn + RVG-IL3 NGs mice. Scale bars, 1 mm for striatum and 500 μm for SNpc. Magnified images of TH-positive cells in the SNpc are shown in the right column (*n* = 7–8 from 3 mice in each group). Scale bars, 50 μm. Results are expressed as mean ± SEM. ^**^*p* < 0.01 versus AAV-GFP; ^##^*p* < 0.01 versus AAV-*h*α-Syn; ^&&^*p* < 0.01, ^&^*p* < 0.05 versus AAV-*h*α-Syn + free IL-3. A one-way ANOVA and a Tukey’s test for *post hoc* comparisons were performed to determine statistical significance.

**
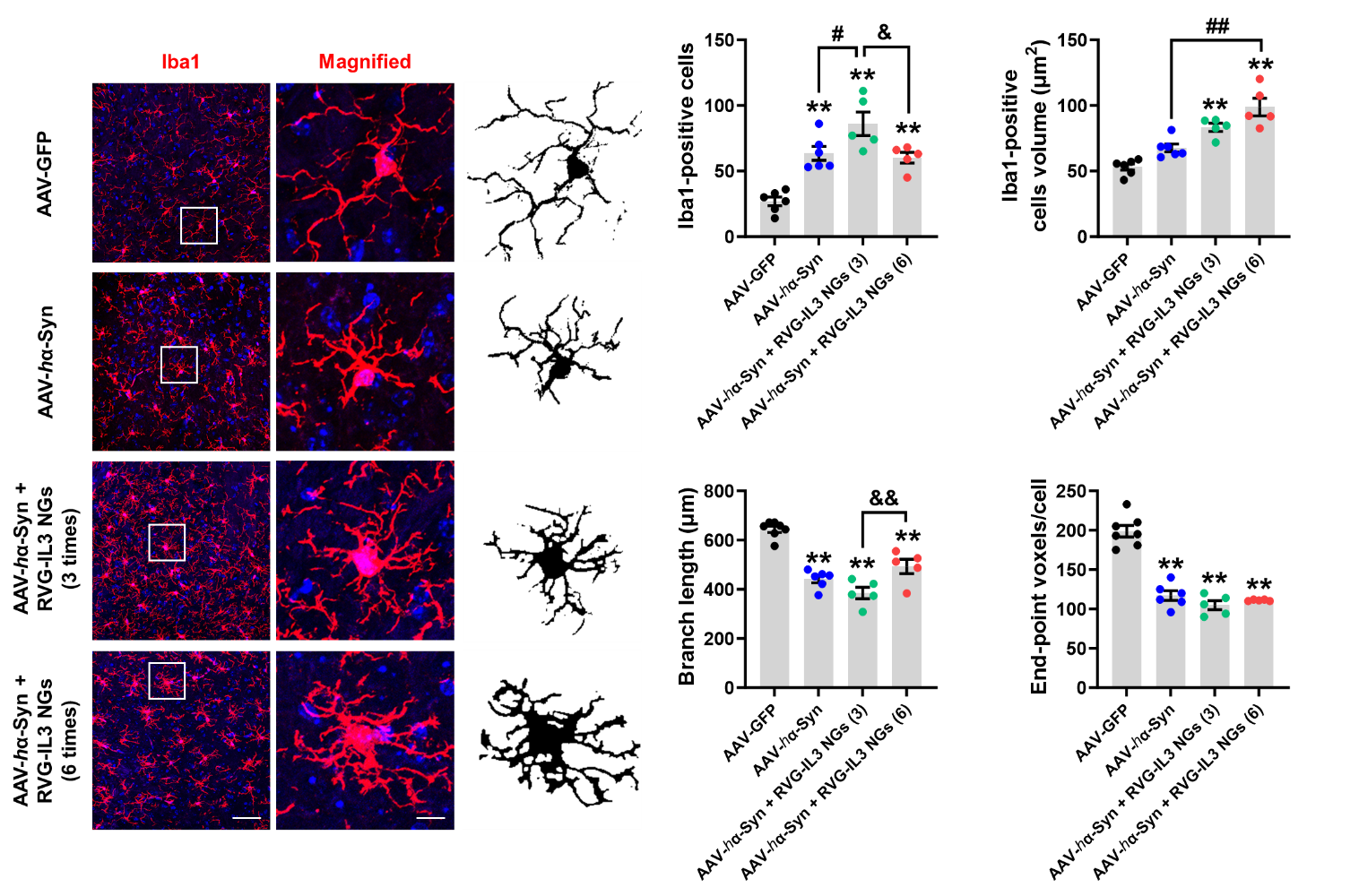
**

**Supplementary Figure S16. Time dependent effect of the RVG-IL3 NGs on the microglial activation in the AAV-*h*α-Syn mice.** Immunostaining and quantification showing the number and volume of Iba1-positive cells in the SNpc. *n* = 5–6 from 3 mice in each group. Scale bar, 50 μm. Magnified images are shown on the right. Scale bar, 10 μm. Results are expressed as mean ± SEM. ^**^*p* < 0.01 versus AAV-GFP; ^##^*p* < 0.01, ^#^*p* < 0.05 versus AAV-*h*α-Syn; ^&&^*p* < 0.01, ^&^*p* < 0.05 versus AAV-*h*α-Syn + RVG-IL3 NGs (treatment for three times). A one-way ANOVA and a Tukey’s test for *post hoc* comparisons were performed to determine the statistical significance.

**
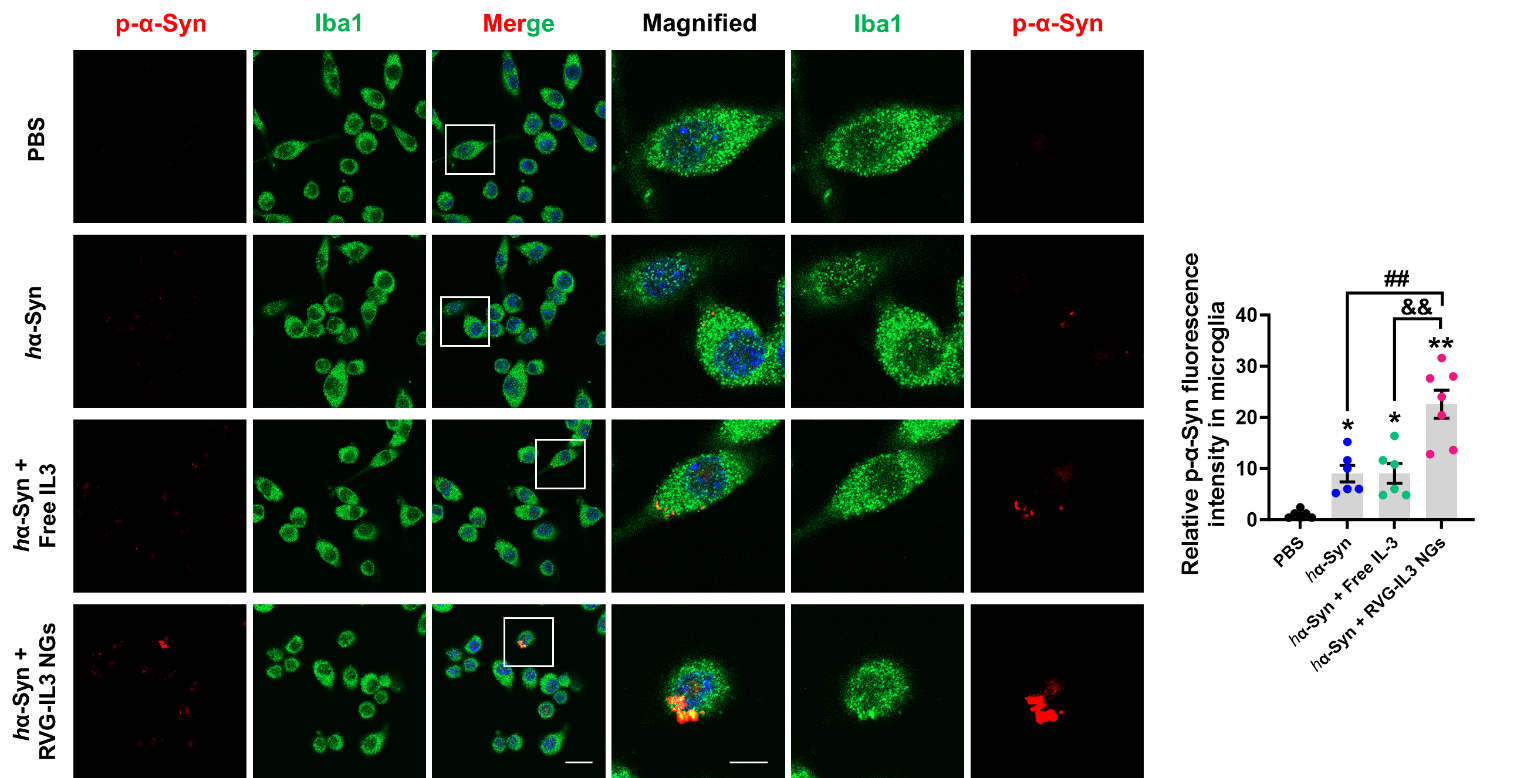
**

**Supplementary Figure S17. Phagocytic pathological α-synuclein by the RVG-IL3 NGs in microglia.** Phagocytic phosphorylated α-synuclein by free IL-3 and RVG-IL3 NGs in the *h*α-Syn fibril-treated microglia. The quantification is shown in the right panel. *n* = 6–7 from 3 mice in each group. Scale bar, 25 μm. Magnified images are shown on the right. Scale bar, 10 μm. Results are expressed as mean ± SEM. ^**^*p* < 0.01, ^*^*p* < 0.05 versus PBS; ^##^*p* < 0.01 versus *h*α-Syn; ^&&^*p* < 0.01 versus *h*α-Syn + free IL-3. A one-way ANOVA and a Tukey’s test for *post hoc* comparisons were performed to determine the statistical significance.

**Supplementary Table S1. Primer sequence used for qRT-PCR.**

| Mice genes | Primer sequence (5’-3’) |
| --- | --- |
| *Pecam1* | F: CACAACAAACAAGCTAGCAAGA  R: TTTGGCTGCAACTATTAAGGTG |
| *Ccr2* | F: GCTCATCTTTGCCATCATGATT  R: TCATTCCAAGAGTCTCTGTCAC |
| *Ccl2* | F: TTTTTGTCACCAAGCTCAAGAG  R: TTCTGATCTCATTTGGTTCCGA |
| *Anxa3* | F: GACCTCGAGGAACCATAAAAGA  R: TTCAAGTCATCTTTCAGCTCCT |
| *Anxa1* | F: GACATTCTTACCAAGAGGACCA  R: GAGCTGGAGTTTTTAGCATAGC |
| *Irgm1* | F: TTTCATCAATGCACTTCGAGTC  R: GTGGGATGAAGAGTACTCAGTC |
| *Adrb2* | F: CACAAAGCCCTCAAGACTTTAG  R: CCTGATAACGTGCACGATATTG |
| *Lix1* | F: CAACTTTCAGTGCTGCTTAAGT  R: TTTGACTCCAGCATGTAGTGAT |
| *Irgm2* | F: CAAAGCTTAGGGAGACACTACA  R: TTTCCGAATCTCTATCGCGTTA |
| *Ifnb1* | F: CTGGGTGGAATGAGACTATTGT  R: AAGTTCCTGAAGATCTCTGCTC |
| *Atg4a* | F: TGGACCAAACACAGTTGCACAGG  R: CAGCAGCACCCACAGGAAGAAC |
| *Gba* | F: GATCCTACTTCTCTACCAACGG  R: AGTTGGATAACTGGAAGTCGTT |
| *Il3ra* | F: CATCCAGAACCTGCATATCGA  R: GAAGACGGTGAAGTTGGTCA |
| *Trp53inp2* | F: TGGAAGGAAATTTGTATCCCGA  R: GTGGATGGTGGTATACTCAGAG |
| *Tcirg1* | F: AGTGAGGAAGGAGTGAGTGCTGTC  R: GTGAAGCGGTTGGTCCTGATGAG |
| *Gapdh* | F: ACGGGAAGCTCACTGGCATGGCCTT  R: CATGAGGTCCACCACCCTGTTGCTG |
